# Genetics and physiology of cell wall polysaccharides in the model C_4_ grass, *Setaria viridis* spp

**DOI:** 10.1186/s12870-015-0624-0

**Published:** 2015-10-02

**Authors:** Riksfardini A. Ermawar, Helen M. Collins, Caitlin S. Byrt, Marilyn Henderson, Lisa A. O’Donovan, Neil J. Shirley, Julian G. Schwerdt, Jelle Lahnstein, Geoffrey B. Fincher, Rachel A. Burton

**Affiliations:** Australian Research Council Centre of Excellence in Plant Cell Walls, School of Agriculture, Food and Wine, University of Adelaide, Waite Campus, Glen Osmond, SA 5064 Australia

**Keywords:** *Cellulose synthase* gene superfamily, (1,3;1,4)-β-glucan, *Setaria*, Immuno-fluorescence microscopy, Q-PCR

## Abstract

**Background:**

*Setaria viridis* has emerged as a model species for the larger C_4_ grasses. Here the *cellulose synthase* (*CesA*) superfamily has been defined, with an emphasis on the amounts and distribution of (1,3;1,4)-β-glucan, a cell wall polysaccharide that is characteristic of the grasses and is of considerable value for human health.

**Methods:**

Orthologous relationship of the *CesA* and Poales-specific *cellulose synthase-like* (*Csl*) genes among *Setaria italica* (*Si*), *Sorghum bicolor* (*Sb*), *Oryza sativa* (*Os*), *Brachypodium distachyon* (*Bradi*) and *Hordeum vulgare* (*Hv*) were compared using bioinformatics analysis. Transcription profiling of *Csl* gene families, which are involved in (1,3;1,4)-β-glucan synthesis, was performed using real-time quantitative PCR (Q-PCR). The amount of (1,3;1,4)-β-glucan was measured using a modified Megazyme assay. The fine structures of the (1,3;1,4)-β-glucan, as denoted by the ratio of cellotriosyl to cellotetraosyl residues (DP3:DP4 ratio) was assessed by chromatography (HPLC and HPAEC-PAD). The distribution and deposition of the MLG was examined using the specific antibody BG-1 and captured using fluorescence and transmission electron microscopy (TEM).

**Results:**

The cellulose synthase gene superfamily contains 13 *CesA* and 35 *Csl* genes in *Setaria*. Transcript profiling of *CslF*, *CslH* and *CslJ* gene families across a vegetative tissue series indicated that *SvCslF6* transcripts were the most abundant relative to all other *Csl* transcripts. The amounts of (1,3;1,4)-β-glucan in *Setaria* vegetative tissues ranged from 0.2% to 2.9% w/w with much smaller amounts in developing grain (0.003% to 0.013% w/w). In general, the amount of (1,3;1,4)-β-glucan was greater in younger than in older tissues. The DP3:DP4 ratios varied between tissue types and across developmental stages, and ranged from 2.4 to 3.0:1. The DP3:DP4 ratios in developing grain ranged from 2.5 to 2.8:1. Micrographs revealing the distribution of (1,3;1,4)-β-glucan in walls of different cell types and the data were consistent with the quantitative (1,3;1,4)-β-glucan assays.

**Conclusion:**

The characteristics of the *cellulose synthase* gene superfamily and the accumulation and distribution of (1,3;1,4)-β-glucans in *Setaria* are similar to those in other C_4_ grasses, including sorghum. This suggests that *Setaria* is a suitable model plant for cell wall polysaccharide biology in C_4_ grasses.

**Electronic supplementary material:**

The online version of this article (doi:10.1186/s12870-015-0624-0) contains supplementary material, which is available to authorized users.

## Background

*Setaria viridis* spp *viridis* (L.) Beauv.SETVI, variously known as wild millet, green foxtail, green millet or green bristlegrass, is the wild ancestor of foxtail millet (*Setaria viridis* spp *italica* SETIT), a widely grown staple grain crop that is prevalent in regions of China, Korea, Japan and India [1]. Both types of millet are found in the Panicoideae subfamily of the order Poales, in the bristle clade of the tribe Paniciae [2]. This “bristle grass” clade includes the economically important C_4_ food crops maize, sorghum, sugarcane and other types of millet, together with species specifically grown as biofuel feedstocks such as switchgrass (*Panicum virgatum*) and *Miscanthus. Setaria viridis* spp, collectively referred to as *Setaria* here, is a self-compatible diploid with a small genome of around 515 Mb but, consistent with its status as one of the most prevalent weeds on the planet [3], it is also small in stature, has a very rapid life cycle of 6–9 weeks and is capable of producing more than 10,000 seeds per plant [4]. Once an *Agrobacterium-*mediated transformation system became established [4, 5] it became clear that *Setaria* would make an excellent model for the much larger, generally polyploid and therefore genetically more complex and intractable C_4_ grasses, and it has been rapidly adopted in this role [6]. In the last few years reference genome sequences of *Setaria* spp. have been released [7, 8] and are accessible from public databases exemplified by Phytozome [9]. Large collections of *Setaria* accessions have been gathered from geographically diverse and ecologically distinct regions of the world. These have facilitated association mapping, allele mining and transcriptomic analysis of traits related to abiotic stress tolerance [10, 11], C_4_ evolution and photosynthesis [12–14], domestication events [15, 16] and biomass production [17]. This explosion of *Setaria*-related resources recently prompted the establishment of the foxtail millet Marker Database (FmMDb) [18]. Foxtail millet is globally the second-most consumed variety of millet behind pearl millet (*Pennisetum glaucum*), which has the major share of the market at 40 % [19]. Millets in general are seen as key crops in many developing countries, where they are cultivated on marginal agricultural land in areas of low rainfall. In these regions, more common cereal crops are not able to grow and millets in their many forms, which include over 100 wild and cultivated species, provide the majority of the energy and protein needs for millions of people, particularly those in sub-Saharan Africa and parts of Asia [20, 21]. In developed countries there is also a resurgent interest in less common grains such as millets because, unlike wheat and related cereals, they contain no gluten. The nutritional composition of generic millets has been defined as being high in starch and protein, although there are relatively low levels of the essential amino acid lysine, and they also contain significant amounts of dietary fibre, calcium and polyphenols [19, 22]. Of particular interest here is the content of non-starchy polysaccharides in green foxtail, but there are only a few reports where these have been examined. In the grain of some cereals there are appreciable levels of polysaccharides, or dietary fibres, which are particularly valuable to human health. These are mainly located in the bran and the endosperm tissues where they are major components of cell walls, which, upon consumption in human diets, undergo hydrolysis and fermentation in the lower digestive tract. Fermentation products include short chain fatty acids, which protect against intestinal disorders and/or colo-rectal diseases [23, 24]. In cereals, the two key polysaccharides in walls of cereal grains are arabinoxylan and (1,3;1,4)-β-glucan [25, 26].

The composition of plant cell walls and the polysaccharides embedded therein has a fundamental effect on human health, but also on the economics and efficient transition of plant biomass to biofuel. Current bioethanol production systems involve the harvesting and conversion to fermentable sugars of lignocellulosic biomasses, either in the form of residues arising from agricultural practices or from purpose-grown crops [27, 28]. The C_4_ grasses maize, sorghum, switchgrass and *Mischanthus* feature prominently in the suite of dedicated bioethanol crops due to attributes such as high yields, growth on marginal lands and drought tolerance [27, 29–31]. In general C_4_ grasses consistently produce higher yields of biomass compared with C_3_ species such as rice, wheat and barley, which are primarily grown as food crops [32]. The raw material that is harvested from the residues of biomass C_4_ crops used for lignocellulosic biofuel production is largely comprised of plant cell walls. There is considerable variation in the composition of the walls in different C_4_ plants and within the individual tissues of these plants [33]. Cellulose is generally the most abundant component of the cell wall in vegetative tissues; it consists of a linear polysaccharide comprised of (1,4)-linked β-glucosyl residues that are readily fermentable once they have been liberated from the polysaccharide. Also present are heteroxylans, which contain (1,4)-linked β-xylosyl residues, together with a range of substituents that are distributed along the xylan backbone, and variable amounts of less abundant polysaccharides that include mannans, pectins and xyloglucans. Relative to hexose sugars, pentose sugars released from these polysaccharides are fermented more slowly. (1,3;1,4)-β-Glucans are present in varying amounts in C_4_ plants, and whilst they are also a linear polysaccharide containing (1,4)-linked β-glucosyl residues, the inherent asymmetry provided by the insertion of (1,3)-linked β-glucosyl residues renders the molecule more soluble than cellulose [34, 35]. Given its more soluble nature and the relative ease with which hydrolytic enzymes can convert it to its component monosaccharides, (1,3;1,4)-β-glucans constitute an ideal source of extractable and fermentable glucose [36]. Although the levels of (1,3;1,4)-β-glucans are generally low in most biomass sources, it has been shown that considerable natural variation exists and that increased levels of the polysaccharide can be engineered through standard genetic manipulation procedures [37, 38]. Deconstructing the cell wall to access the monosaccharides, glucose in particular, can be expensive and complex because of the recalcitrant nature of cellulosic biomass [39–41]. The presence of lignin contributes to this recalcitrance, because lignin forms an interlinked network around the polysaccharide components. The accessibility of cell wall polysaccharides to hydrolytic enzymes is an important economic consideration in the conversion of biomass to bioethanol. The hydrolysis of different polysaccharides often requires pre-treatment of the biomass and at least three different types of enzymes, and the cost of these steps may be a large proportion of the overall budget of the ethanol production process [42].

The general cell wall composition of *S. viridis* has been analysed and compared with the other C_4_ crops maize, sorghum and switchgrass [17]. The major components of the cell walls such as cellulose, lignin and neutral sugars have been reported. However, neutral sugars were used as a collective measurement of ‘hemicelluloses’ and specific levels of (1,3;1,4)-β-glucans were not quantitated [17]. Here, the phylogeny of gene families comprising the *cellulose synthase* superfamily in *Setaria* is defined, with a focus on the genetics and transcription of *cellulose synthase-like* genes that encode the synthases responsible for the production of (1,3;1,4)-β-glucan. The amount, structure and distribution of (1,3;1,4)-β-glucan in the tissues of *S. viridis* is reported, enabling this species to be assessed in the context of the value of its grain to human health and its utility as a model for other C_4_ grasses in a biofuel context.

## Results

### The *cellulose synthase* superfamily in *Setaria*

#### *Cellulose synthase* genes (*CesA*)

The orthologous relationships of the *CesA* genes between *Setaria italica*, barley (*Hordeum vulgare*), sorghum (*Sorghum bicolor*), *Brachypodium distachyon* and rice (*Oryza sativa*) are represented on a Bayesian phylogenetic tree (Additional file 1: Figure S1). The analysis indicates that there are 13 *cellulose synthase* (*CesA*) genes in total in *S. italica*, which revises the previous estimate of eight reported by Petti *et al.* [17]. The genes are spread across five of the nine chromosomes (Additional file 2: Table S1) and they have been numbered according to their barley orthologues [43]. There are single representatives of *CesAs 1, 3, 4, 5, 8* and *10* genes with no corresponding orthologues of the barley *CesA7* or *CesA9* genes [44]. There are four and three genes that are very closely related to *HvCesA2* and *HvCesA6*, respectively. *SiCesA2-1* is on chromosome 4, whereas *SiCesA2-2* and *SiCesA2-3* are located on chromosome 2 and *SiCesA2-4* is on chromosome 9. Each of the three *CesA6* paralogues are distributed on different chromosomes; *SiCesA6-1* on chromosome 5, *SiCesA6-2* on chromosome 4 and *SiCesA6-3* on chromosome 3.

#### *Cellulose synthase-like* genes (*Csl*)

**Fig. 1:**
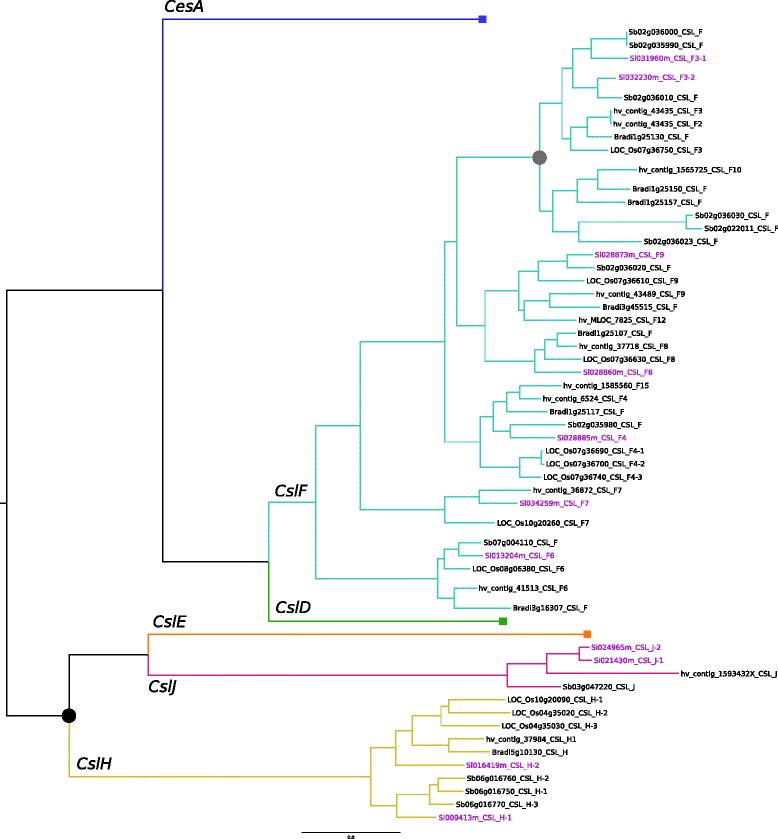
Orthologous relationship of the *Cellulose synthase-like* (*Csl*) genes. *Setaria italica (Si)*, *Sorghum bicolor (Sb)*, *Oryza sativa (Os)*, *Brachypodium distachyon* (*Bradi*) and *Hordeum vulgare (Hv)* are compared. Branch lengths are proportional to nucleotide substitutions per site. Black dot node labels indicate posterior probability of 0.6-0.85, whilst grey dots indicate a posterior probability of 0.85-0.95, and unlabelled nodes present a posterior probability of >0.95

In *S. italica* a total of 35 *cellulose synthase-like* (*Csl*) genes have been identified, a sub-set of which are displayed on the Bayesian phylogenetic tree in Additional file 1: Figure S1. There are eleven *CslA*s and six *CslC*s (data not shown), which represent the more basal clades of this superfamily, together with five *CslD*, two *CslE*, seven *CslF*, two *CslH* and two *CslJ* genes (Additional file 1: Figure S1). Of the *cellulose synthase-like* gene families restricted to the Poaceae, *Setaria* is unlike barley, rice or sorghum with respect to *CslH*s. It has two members of this family whilst barley and *Brachypodium* have one, and rice and sorghum both have three (Fig. 1). *Setaria* also carries two distinct *CslJ* genes whereas all other grasses analysed to date have either none or just a single *CslJ* gene (Fig. 1). As expected, there are no representatives in the dicot-specific *CslB* and *CslG* families.

The *CslF* family, with only seven members, is smaller than the barley family, which has eleven members [45, 46]. Like barley there are no equivalent *Setaria* orthologues to the *CslF1* and *CslF2* genes of rice [45] and there are single genes corresponding to *CslF4, 6, 7, 8* and *9* (Fig. 1)*.* Genes matching barley *CslF10, 11, 12* and *13* [46] are also absent, but there appears to have been a recent duplication of the *CslF3* gene in *Setaria* with two copies arranged as a tandem repeat on chromosome 2 (Additional file 2: Table S1). This is on the end of a gene cluster that also includes *SiCslF4, 8* and *9*.

All of the *SiCslD* genes are found on different chromosomes, and the two *SiCslH* genes are also separated onto chromosomes 7 (*SiCslH1)* and 1 (*SiCslH2).* However, the small sets of *SiCslE* and *SiCslJ* genes possibly arise from recent duplication events because they are present as adjacent pairs on chromosomes 2 and 3, respectively (Additional file 2: Table S1).

### Transcript profiling of *cellulose synthase-like* genes

**Fig. 2:**
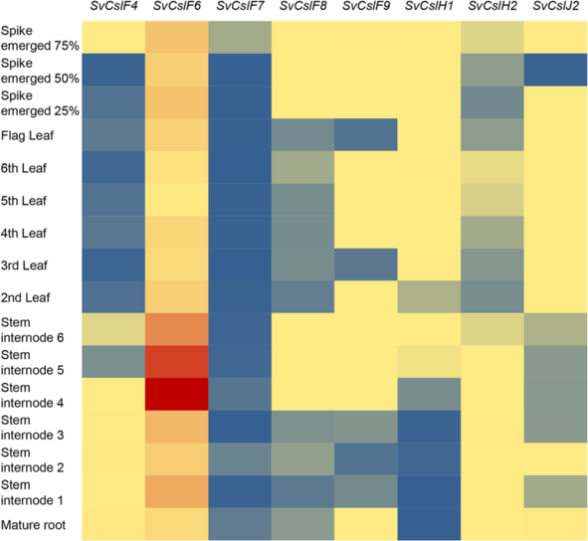
Heat map of *Cellulose synthase-like* gene transcripts from *S. viridis* vegetative tissues*.* The gene expression level is indicated by red, yellow and blue for high, medium and low expression, respectively

**Fig. 3:**
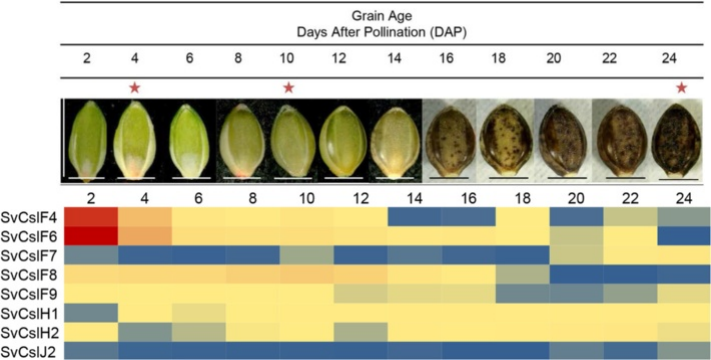
Heat map of *Cellulose synthase-like* gene transcripts from *S. viridis* developing grain*.* The gene expression level is indicated by red, yellow and blue for high, medium and low expression, respectively. Asterisk: grain was sectioned in different stages for (1,3;1,4)-β-glucan distribution analysis, i.e. young (4 DAP); intermediate (10 DAP); mature (24 DAP). Magnification: 125 x. Bars: horizontal bar 1.5 mm; vertical bar 2 mm

The genome sequence of the model plant *Setaria* is publicly available and is largely accounted for by reads from *S. italica* [8]. This was used as a reference in order to identify putative *cellulose synthase-like* sequences in the closely related *S. viridis* [8]. Primers were designed to the predicted 3’ untranslated region of the *S. viridis* genes of the *CslF, H* and *J* clades (Additional file 2: Table S2) and used to examine the transcript levels by real-time quantitative PCR (Q-PCR) across a *S. viridis* tissues series (Fig. 2). Within the *CslF* family *SvCslF6* transcripts were present at relatively high levels in all tissues examined. Transcript levels were highest in RNA from stem internode 4, at around one million copies (Additional file 1: Figure S2). The transcripts of *SvCslF8* and *SvCslF9* were the next most abundant in tissues that have not reached full maturity, such as leaves and younger stem internodes (Additional file 1: Figure S2). However, in the more mature root and stem tissues, *SvCslF4* transcripts were present at levels above those of *SvCslF8* and *9* (Additional file 1: Figure S2). *SvCslF6* and *SvCslF4* transcripts were relatively high in the grain development series, where they started at high levels at 2 DAP but dropped rapidly to very low levels by 6 DAP (Fig. 3, Additional file 1: Figure S2).

Close examination of the transcript levels of the two *SvCslH* genes showed that *SvCslH1* transcripts were significantly higher in leaf and spike tissues, which were still growing at the time of harvest. In contrast, *SvCslH2* transcripts were high in older stem internodes and in mature roots (Additional file 1: Figure S3). This suggests there may be a negative correlation between the abundance of the two transcripts (Additional file 1: Figure S4). A positive correlation was observed between the transcript levels of *SvCslF4* and *SvCslH2* across vegetative tissues (Additional file 1: Figure S4). Transcript levels of both *SvCslH1* and *2*, and *CslJ2* were negligible during grain development (Additional file 1: Figures S3 and S5). The QPCR data indicated that *SvCslJ2* transcripts were present at significantly high levels in the mature root and the older inflorescence, second in magnitude only to *SvCslF6* (Additional file 1: Figure S5).

### Abundance and fine structure of (1,3;1,4)-β-glucan in vegetative tissues

**Fig. 4:**
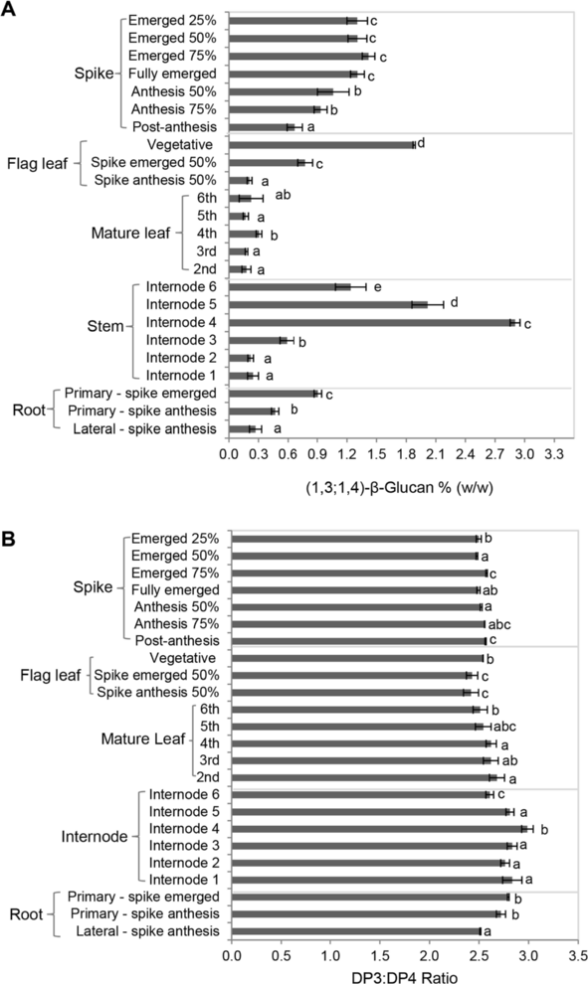
(1,3;1,4)-β-Glucan content and DP3:DP4 ratios in *S. viridis* vegetative tissues. **a** Mean amounts of (1,3;1,4)-β-glucan in vegetative tissues. The values vary significantly according to the developmental stage of the tissue. **b** DP3:DP4 ratios in vegetative tissues. Error bars indicate standard deviation (*n* = 4). *T*-test compared differences within the same group. Tissues with the same letter indicate non-significant differences (*P* > 0.05); different letters indicate significant differences (*P* < 0.05)

The amount of (1,3;1,4)-β-glucan and the ratio of cellotriosyl to cellotetraosyl units (DP3:DP4 ratio) in the vegetative tissues of *S. viridis* were quantitated at different stages of plant development from vegetative-leaf to reproductive-floral stages (Additional file 1: Figure S11). The amount of (1,3;1,4)-β-glucan changed during plant development and generally decreased as tissues matured (Fig. 4a). For example, the amount in the flag leaf decreased from 1.9 % (w/w) at the vegetative-leaf stage to 0.2 % (w/w) at anthesis (Fig. 4a). Similarly, the amount in the primary (seminal) root decreased from 0.9 % (w/w) at the early reproductive-floral stage to 0.5 % (w/w) at anthesis (Fig. 4a), whilst in the inflorescence, the amount decreased from 1.3 % (w/w) to 0.7 % (w/w) over the same time frame (Fig. 4a).

However, there was a different trend in the amount of (1,3;1,4)-β-glucan found in the stem, where the youngest stem internode did not contain the most (1,3;1,4)-β-glucan. At anthesis the highest amount of (1,3;1,4)-β-glucan was observed in internode 4 (2.9 % w/w, Fig. 4a). The youngest (internode 6) and oldest (internode 1) stem internodes contained 1.2 % (w/w) and 0.2 % (w/w) (1,3;1,4)-β-glucan, respectively. The amount of (1,3;1,4)-β-glucan also varied significantly (*P* < 0.05) in different root tissues (Fig. 4a). For example, at anthesis the lateral (adventitious) root contained 0.3 % (w/w) (1,3;1,4)-β-glucan, less than the 0.5 % (w/w) in the primary (seminal) root. However, the amounts in different leaves, when harvested at the mature plant stage (Fig. 4a), were similar at 0.2–0.3 % (w/w). The DP3:DP4 ratios across the *S. viridis* vegetative tissue samples ranged from 2.4:1 to 3.0:1 (Fig. 4b). The lowest ratio, 2.4:1, was in the flag leaf at the late reproductive-floral stage, and this ratio was significantly lower (*P* < 0.05) than the ratio of 2.7:1 that was measured in the oldest leaf (Fig. 4b). The highest ratio of 3:1 was observed in internode 4, and this ratio was significantly higher (*P* < 0.05) than the ratio in all other internodes (Fig. 4b).

### Abundance and fine structure of (1,3;1,4)-β-glucan in developing grain

The amounts of (1,3;1,4)-β-glucan and the DP3:DP4 ratios in *S. viridis* grain were measured across a grain developmental series (Table 1, Fig. 3). Very young grains were pooled from 2 days after pollination (DAP) to 6 DAP, while intermediate and older stage grains were harvested at 8–14 DAP and 16–24 DAP, respectively. The amount of (1,3;1,4)-β-glucan decreased from 0.013 % (w/w) in the youngest caryopsis to 0.003 % (w/w) as the grain matured (*P* < 0.05, Table 1). The DP3:DP4 ratios in the young (2.7:1), intermediate and mature grain stages did not differ significantly (2.5:1 compared with 2.8:1, Table 1).

**Table 1:** Mean amounts of (1,3;1,4)-β-glucan and DP3:DP4 ratios in *S. viridis* developing grain

Age (DAP)	Percentage (w/w)^d^	DP3:DP4Ratio (x:1)^d^
2 – 6 (young)	0.013 ± 0.0006^a^	2.7 ± 0.18^ab^
8 – 14 (intermediate)	0.004 ± 0.0005^b^	2.8 ± 0.05^a^
16 – 24 (mature)	0.003 ± 0.0003^c^	2.5 ± 0.05^b^

Values are means ± standard deviation measured using the Megazyme assay (*n* = 3) and HPAEC-PAD. ^d^Grain with different superscript letters is significantly different (*P* < 0.05) GenStat 15^th^ Ed. SP2

### Distribution of (1,3;1,4)-β-glucan in the leaves

**Fig. 5:**
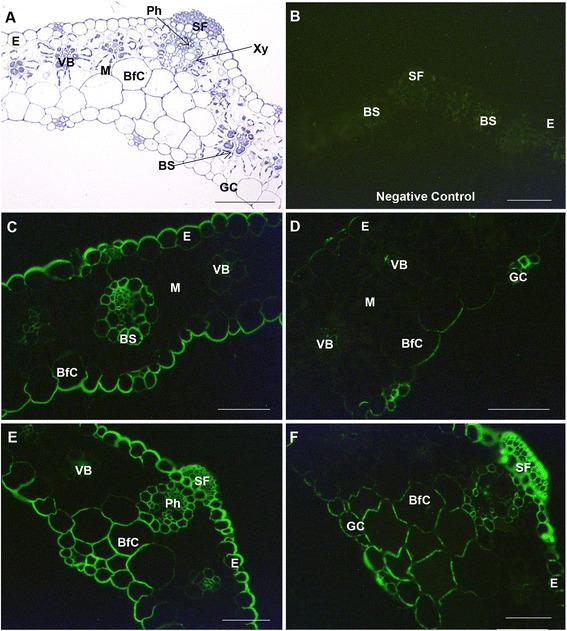
Micrographs of leaf transverse sections. **a** Bright-field light micrograph of a toluidine blue-stained survey section. **b**-**f** Fluorescence light micrographs using **b** an absent of primary and secondary antibodies as negative control, **c**-**f** an antibody conjugated to Alexafluor 488. (1,3;1,4)-β-Glucan is indicated by green fluorescence. **c** Young leaf blade. **d** Old leaf blade. **e** Young leaf across midrib. **f** Old leaf across midrib. E, epidermis; BS, bundle sheath; M, mesophyll; Xy, xylem; Ph, phloem; SF, sclerenchyma fibre; GC, guard cells; VB, vascular bundle; BfC, bulliform cells. Magnifications: **a**, **e** 200 x; **b**–**d**, **f** 400x. Bars: **a**, **e** 100 μm, **b**–**d**, **f** 50 μm. Exposure time: **b**–**f** 1.6 s

**Fig. 6:**
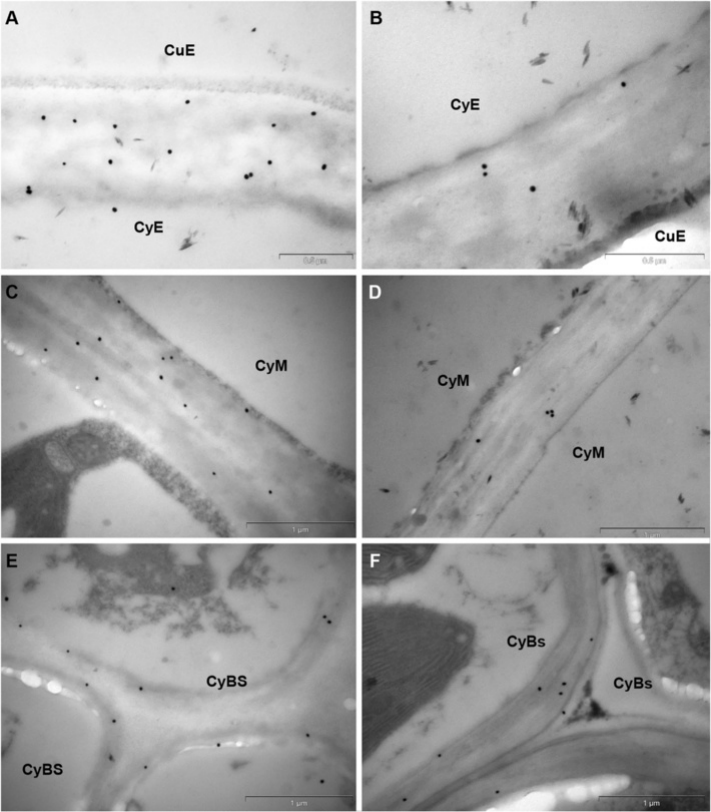
TEM micrographs of leaves labelled with BG-1. Walls of epidermal cells in **a** young and **b** mature leaves. Walls of mesophyll cells in **c** young and **d** mature leaves. Walls of contiguous bundle sheath cells in **e** young and **f** mature leaf. CuE, cuticle of epidermal cell; CyE, cytoplasm of epidermal cell; CyM, cytoplasm of mesophyll cell; CyBS, cytoplasm of bundle sheath cell. Gold labelling: 25 nm gold particles. Scale bars: **a**, **b** 0.5 μm; **c**-**f** 1 μm

The typical C_4_ Kranz anatomy of the *S. viridis* leaf was evident in toluidine blue section (Fig. 5a). The distribution of (1,3;1,4)-β-glucan in *S. viridis* leaves at different developmental stages was captured using fluorescence microscopy and immunolabelling with specific antibodies for (1,3;1,4)-β-glucans. The micrographs indicated differences in (1,3;1,4)-β-glucan distribution between the younger leaf at inflorescence emergence (IE) stage and the older leaf at anthesis stage. Immunolabelling of the younger leaf was heavier than that of the older leaf (Fig. 5c vs. d, Fig. 5e vs. f). In the flag leaf at IE, (1,3;1,4)-β-glucans were distributed in the walls of every cell type (Fig. 5c), whilst in the older leaf they were concentrated mostly in the walls of cells in the midrib area, including schlerenchyma fibres, bundle sheath cells, bulliform and guard cells (Fig. 5d, f). Higher resolution immunocytochemical examination of (1,3;1,4)-β-glucans in the epidermal cell walls using transmission electron microscopy (TEM) also showed a higher density of labelling in the young leaf compared with the old leaf (Fig. 6a). In the young leaf, labeled (1,3;1,4)-β-glucans were detected throughout the walls, whilst in the old leaf, they were sparsely distributed (Fig. 6b, Additional file 1: Figure S6). The (1,3;1,4)-β-glucans in cell walls that showed limited fluorescence, such as in the mesophyll cells (Fig. 5c-d), were examined using the more sensitive TEM (Fig. 6c-6d, Additional file 1: Figure S6). In general, detection of the (1,3;1,4)-β-glucan in the young leaf by gold labelling was consistent with the fluorescence labelling results, including the distribution of (1,3;1,4)-β-glucan in the bundle sheath cells (Fig. 6e). TEM micrographs also indicated that (1,3;1,4)-β-glucans were detected in the walls of the bundle sheath cells in the midrib area of the older leaf (Fig. 6f, Additional file 1: Figure S6).

### Distribution of (1,3;1,4)-β-glucan in the stem

**Fig. 7:**
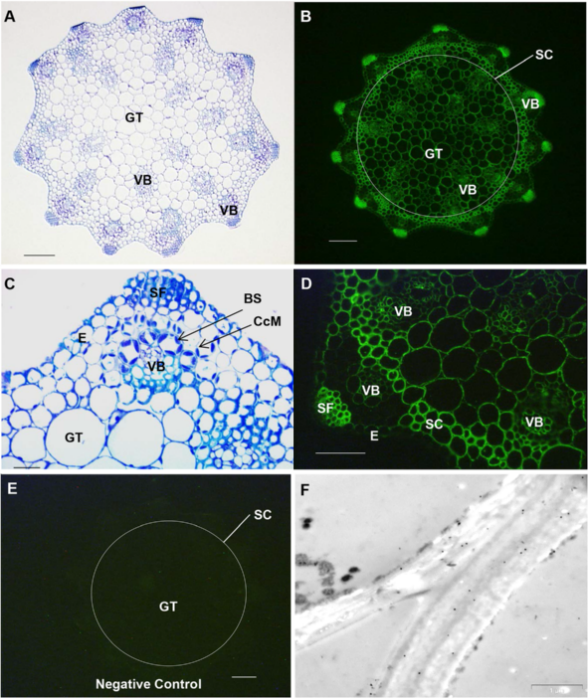
Micrographs of stem transverse sections. **a**, **c** Bright-field light micrographs of a toluidine blue-stained survey section. **b**, **d** Fluorescence light micrographs using an antibody conjugated to Alexafluor 488 where (1,3;1,4)-β-Glucan is indicated by green fluorescence. **e** Fluorescence light micrograph with a secondary antibody only as negative control. **f** TEM micrograph of walls of ground tissue cell. E epidermis; BS bundle sheath; CcM Chloroplast-containing mesophyll; SF, sclerenchyma fibre; SC, sclerenchyma cylinder, GT, ground tissue; CyGT, cytoplasm of ground tissue cell; VBW, vascular bundle wall. Arrows indicate gold labelling. Magnifications: **a**, **b**, **e** 100 x; **c**, **d** 400 x. Scale bars: **a**, **b**, **e** 100 μm; **c**-**d** 50 μm; **f** 1 μm. Exposure time: **b**, **d**, **e** 2.6 s

The *S. viridis* stem has a typical monocot anatomy, with a sclerenchyma cylinder of vascular bundles embedded in chloroplast-containing mesophyll tissue [47], and this was evident in toluidine blue-stained sections (Fig. 7a, c). In fluorescence micrographs, there was stronger (1,3;1,4)-β-glucan labelling in the sclerenchyma fibre cells than in the surrounding mesophyll cells of the stem rind area (Fig. 7b, d). In the inner pith area, the labelling indicated an even distribution of the polysaccharide in all cell walls, including in the two rings of vascular bundles and in the ground tissue cells (Fig. 7b). TEM micrographs showed that there was more (1,3;1,4)-β-glucan in the walls of ground tissue cells compared with those of the vascular bundles (Fig. 7, Additional file 1: Figure S7).

### Distribution of (1,3;1,4)-β-glucan in the root

**Fig. 8:**
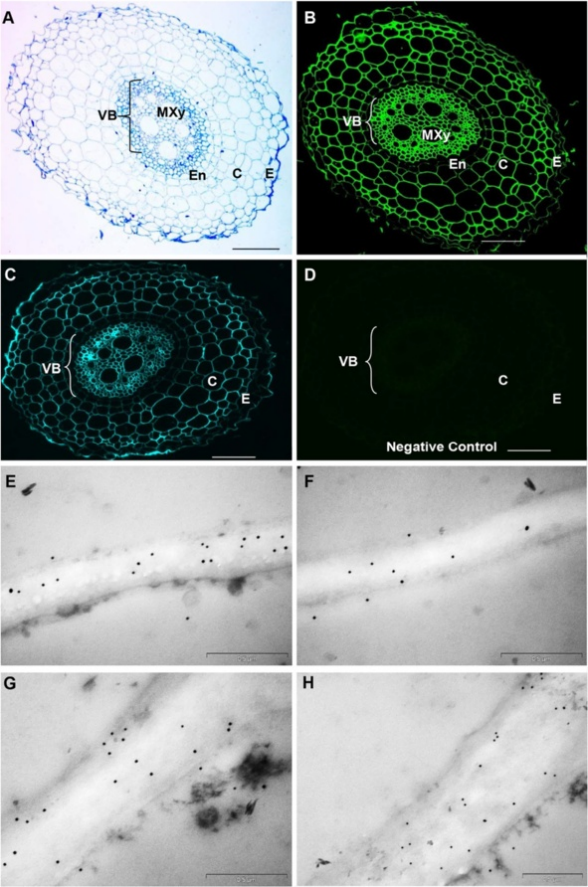
Micrographs of main root transverse sections. **a** Bright-field light micrograph of a toluidine blue-stained survey section. **b**-**d** Fluorescence light micrographs using **b** an antibody conjugated to Alexafluor 488, **c** Calcofluor White MR2, **d** a secondary antibody only as negative control. (1,3;1,4)-β-Glucan is indicated by green fluorescence. **e**-**h** TEM micrographs of sections immunogold labelled with the (1,3;1,4)-β-glucan antibody BG-1. Walls of cells in the **e** epidermis, **f** cortex, **g** endodermis and **h** vascular bundle. E, epidermis; C, cortex; En, endodermis; VB, vascular bundle; Mxy, metaxylem. Magnification: **a**-**d** 100 x. Scale bars: **a**-**d** 100 μm; **e**-**h** 0.5 μm. Exposure time for **b** = 650 ms

The anatomy of the main root of *S. viridis* was represented in a toluidine blue-stained section (Fig. 8a). Fluorescence micrographs of immunolabelled roots indicated that (1,3;1,4)-β-glucans were distributed evenly across all cell types except in the walls of endodermal cells (Fig. 8b). However, the more sensitive TEM and immunogold labelling procedures revealed the presence of some (1,3;1,4)-β-glucans in the walls of the endodermal cells (Fig. 8g, Additional file 1: Figure S8).

### Distribution of (1,3;1,4)-β-glucan in *Setaria* grain

**Fig. 9:**
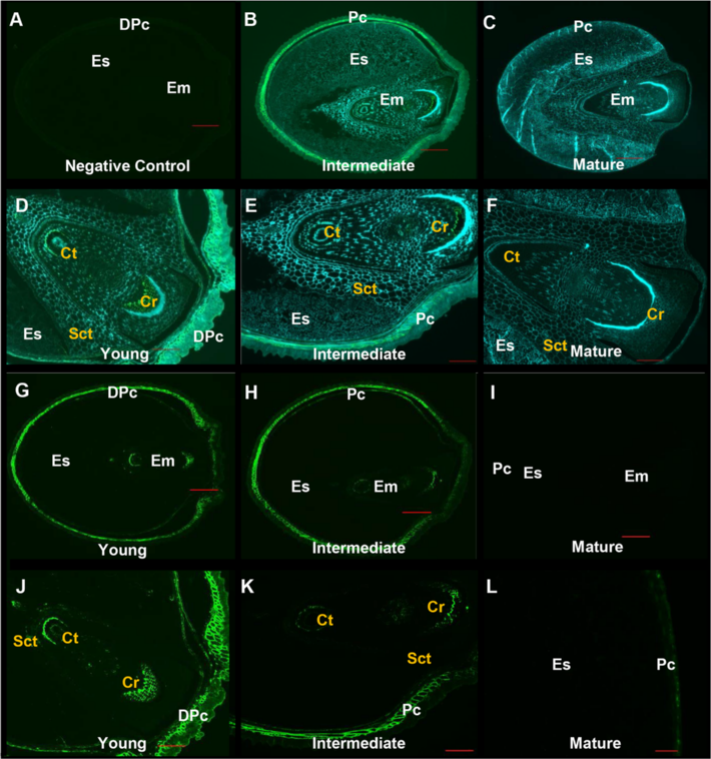
Micrographs of developing grain transverse sections. **a**-**f** Combination fluorescence light micrographs showing overlaid signals from both an antibody conjugated to Alexafluor 488 and Calcofluor White MR2. Fluorescence light micrographs using **a** a primary antibody only as negative control, **g**-**l** an antibody conjugated to Alexafluor 488 where (1,3;1,4)-β-glucans are indicated by green fluorescence. DPc, developing pericarp; Em, embryo; Sct, scutellum; Ct, coleoptile; Cr, coleorhiza; Es, endosperm. Magnification: **a**-**c**, **g**-**i** 5 x; **d**-**f**, **j**, **k** 100x; (**l**) 40x. Scale Bars: **a**-**c**, **g**-**i** 200 μm; **d**-**f**, **j**, **k** 100 μm; 20 μm. Exposure time: **a**-**c**, **g**-**i** Alexafluor 488 = 860.4 ms, Calcofluor White MR2 = 700 ms; **d**-**f**, **j**, **k** Alexafluor 488 = 550, Calcofluor White MR2 = 260 ms; **l** Alexafluor 488 = 100 ms, Calcofluor White MR2 = 600 ms

**Fig. 10:**
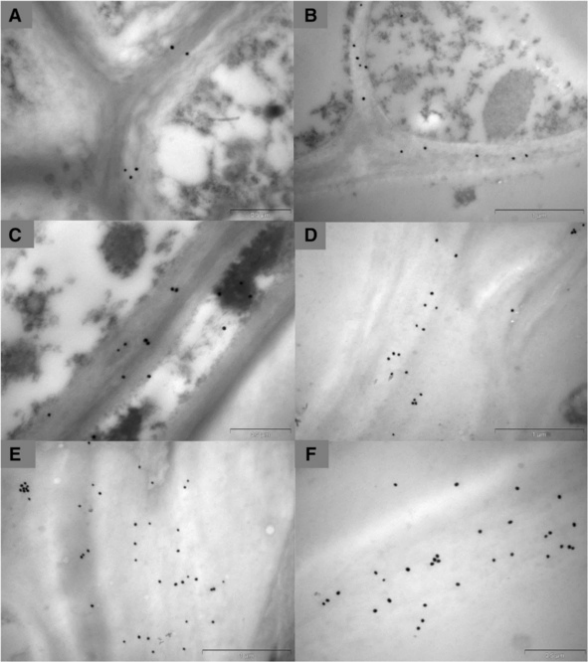
TEM micrographs of grain labelled with BG-1. **a** Cell walls of the embryo in the mature grain. Walls of the aleurone in the **b** young and **c** mature grain. Walls of the pericarp in the **d** young, **e** intermediate and **f** mature grain. Gold particles: **a**-**f** 25 nm. Scale bars: **a**, **c** 0.5 μm; **b**, **d**, **e**, **f** 1 μm

The phenotype of the *S. viridis* grain during development was observed from 2 DAP to 24 DAP (Fig. 3). Three different stages, namely young (4 DAP), intermediate (10 DAP) and mature (24 DAP), were sectioned to map the distribution of (1,3;1,4)-β-glucan (Fig. 9). Fluorescence micrographs of Alexa Fluor® 488 and Calcoflour-White MR2 labelling represented the anatomical structure of cell walls in the grain in transverse sections (Fig. 9a–f), which was consistent with previous reports of *S. viridis* anatomy by Winton and Winton [48] and of *Setaria lutescens* by Rost [49]. A higher magnification micrograph of the outer layer of the young grain displayed a developing pericarp consisting of different layers, namely the cuticle, cross cells, tube cells, testa and nucellus (Additional file 1: Figure S9A). The pericarp was fully developed in the intermediate grain (Additional file 1: Figure S9B). The pericarp surrounds the endosperm, which was comprised of aleurone cells and the starchy endosperm, and the scutellum of the embryo (Additional file 1: Figure S9B). Higher magnification micrographs (Fig. 9d–f) showed the typical embryonic structure of *Setaria* grain. The scutellum forms a cuplike structure that surrounds the axis of the coleoptile and coleorhiza [49, 50]. Fluorescence micrographs indicated that (1,3;1,4)-β-glucans were most abundant in the pericarp and embryo of the younger grain (Fig. 9g, j) particularly in the coleoptile and coleorhiza. Lower levels of (1,3;1,4)-β-glucans were present in the intermediate embryo (Fig. 9h, k) and (1,3;1,4)-β-glucans were almost completely absent in the pericarp and the mature embryo of the oldest grain (Fig. 9i, l). However, the use of TEM confirmed the presence of some (1,3;1,4)-β-glucans in cell walls where they were not clearly detected by fluorescence immunolabelling, such as in the embryo of the mature grain (Fig. 10a) or in the scutellum of the young and intermediate grain (Additional file 1: Figure S10). Labelling of (1,3;1,4)-β-glucan in the aleurone walls of the young (Fig. 10b), intermediate (Additional file 1: Figure S10) and mature grain (Fig. 10c) was also observed. In line with the fluorescence results, (1,3;1,4)-β-glucan in the walls of the pericarp was detected throughout development using TEM (Fig. 10d–f, Additional file 1: Figure S10).

## Discussion

### Genes in the *Setaria cellulose synthase* superfamily

There are at least 48 genes in the *cellulose synthase* superfamily in *Setaria* (Fig. 1 and Additional file 1: Figure S1), which is consistent with most other land plants. Of these, 13 are *CesA*s, five more than the eight previously reported by Petti et al. [17] and similar to numbers in other cereals in the Poales, where barley and rice have nine [43, 44, 51], sorghum has 10 [52], and more broadly to other flowering plants where Arabidopsis also has 10 [53].

The *CesA* genes are distributed over five of the nine chromosomes and do not appear to cluster (Additional file 2: Table S1, Additional file 1: Figure S1). In *Setaria*, *CesA4* and *CesA8* resolve as the ancestral lineages, while the phylogeny suggests that *CesA5* was the next group to split, followed by a large diverse clade that contains genes corresponding to *CesA1* and *CesA3*, *CesA2* (4 genes) and *CesA6* (3 genes). The *CesA10* gene is grass specific and belongs to a highly divergent group that has lost the QXXRW catalytic motif [52]. It is not clear why there are so many closely related *CesA* genes in *Setaria* and why the *CesA2* and *CesA6* genes, associated with synthesis of cellulose in primary cell walls [43], have duplicated more than once in recent evolutionary history. The larger *CesA* gene family will make the identification of encoded proteins that participate in the formation of the classical heteromeric terminal rosette complexes, and the definition of their levels of redundancy, particularly difficult to unravel. The proposed participation of two sets of three different *CesA* genes in barley is based on co-transcription in tissues where primary or secondary wall synthesis predominates [43]. No such correlation could be detected in our transcript profiles from many tissues of *Setaria* (Additional file 1: Figure S2).

*Setaria* lacks a few members of the *CslF* family including orthologues of the most recently identified members in barley, namely *CslF10*, *11, 12* and *13,* and the *CslF1* and *CslF2* genes of rice [45, 46]. Not withstanding the ‘missing’ genes, the seven *CslF*s are organised across the genome akin to the family in other grasses with a syntenic main cluster on chromosome 2 ([52], Additional file 2: Table S1) and the remaining members are scattered on individual chromosomes. The exception are the two closely related genes *SiCslF3-1* and *SiCslF3-2*, found next to each other on chromosome 2, at one end of the *CslF* cluster and which appear to be the result of recent gene duplication.

*Setaria* has two genes in each of the *CslH* and the *CslJ* families, while sorghum and rice have three *CslH* genes, sorghum has a single *CslJ* (Fig. 1) and barley has a single member of both families [52, 54]. The two *SiCslH* genes are separated on chromosomes 1 and 7 (Additional file 2: Table S1). This is the first report of a cereal that carries two *CslJ*s where the second one derives from a gene duplication event because the two copies share 87.3 % identity at the amino acid level.

### The *SvCslF6* gene is highly transcribed

The *CslF6* transcript is dominant in the majority of tissues of most C_3_ and C_4_ cereals and the corresponding CSLF6 protein is likely to be the major synthase driving the deposition of (1,3;1,4)-β-glucan [37, 38, 45, 55–57]. *Setaria* is not an exception to this, since the *CslF6* transcript is found at the highest levels of all the *CslF, H* and *J* genes in vegetative tissues at all stages of development examined here. For example, in the elongating fourth stem internode *SvCslF6* transcripts peaked at around one million copies per microliter and in tissues where expansion has ceased, such as the mature root (Additional file 1: Figure S2) approximately 100,000 copies of *SvCslF6* per microliter were detected. The temporal and spatial occurrence of (1,3;1,4)-β-glucan observed is similar to those described for other C_4_ grasses, including maize and sorghum [58–61]. In general the amount of (1,3;1,4)-β-glucan in *Setaria* decreases during maturity in roots, leaves and the inflorescence and levels vary significantly according to the age of the tissues (Fig. 4a).

The physiological role of (1,3;1,4)-β-glucan in plants is debated in the literature [59, 62–65]. The decrease in the amount of (1,3;1,4)-β-glucan in leaves, roots and stem sections as they age indicates that it may be more important in developing tissues than mature ones; in the roots it decreased from 0.9 % w/w at inflorescence emergence to 0.5 % (w/w) at anthesis (Fig. 4a) possibly linked to tiller production, as grasses stop developing their primary (seminal) roots when mature tillers become established [66]. Similar trends have been reported for other C_4_ grasses. In maize, sorghum and barley (1,3;1,4)-β-glucan is synthesised in seedlings and coleoptiles, leaves and stems when elongation starts and ceases when elongation stops [60, 61].

A role for (1,3;1,4)-β-glucan in carbon storage in barley and sorghum vegetative tissues has been proposed [62–65]. The change from 1.9 % (w/w) (1,3;1,4)-β-glucan in the flag leaf during vegetative growth to 0.3 % (w/w) at anthesis could indicate that (1,3;1,4)-β-glucan may be hydrolysed at this stage to remobilise carbon reserves [62] and this may explain the presence of (1,3;1,4)-β-glucan reserves in the *Setaria* stem. The largest amount of (1,3;1,4)-β-glucan was observed in stem internode 4 (2.95 % w/w, Fig. 4a) and TEM labelling indicated that these reserves were more highly concentrated in ground tissue walls relative to those of the vascular tissue (Additional file 1: Figure S7). Mobilisation of this secondary carbon reserve may be particularly important to *S. viridis*, because it has such a rapid life cycle and produces a great many seeds.

The properties of (1,3;1,4)-β-glucans may be linked to their fine structure, as determined by the DP3:DP4 ratio [67, 68]. A lower ratio is generally associated with higher solubility of the polysaccharide and this property is of interest in a human health context [69] and may affect ease of extraction for bioethanol production from lignocellulosic feedstocks [37, 70]. There is evidence that individual CSLF enzymes synthesize (1,3;1,4)-β-glucans with different fine structures [36, 71]. Subtle but significant differences in the (1,3;1,4)-β-glucan DP3:DP4 ratios in a range of different *Setaria* tissues were measured. Where (1,3;1,4)-β-glucan may be structurally important, such as in the actively growing parts of the plant (primary roots and stem internode 4, the ratio is relatively high. However, in tissues where cell growth slows or ceases and the remobilisation of carbon reserves is likely to occur, the ratio and solubility of (1,3;1,4)-glucans might be expected to be lower. This was observed in some tissues where remobilisation might be occurring, for example in mature stem internodes, but the correlation was not consistent.

The activity of *CslH* genes could significantly influence the amount and nature of (1,3;1,4)-β-glucan and thus the overall cell wall properties. Transcript levels of the barley *HvCslH1* and the sorghum *SbCslH3* genes were reported to be higher in mature tissues, such as the leaf tip, relative to young tissues [54, 61]. This trend was also observed for the *Setaria CslH2* transcript. The peak transcripts of the *CslH* gene have already been linked to the presence of (1,3;1,4)-β-glucan with a higher DP3:DP4 ratio, and thus lower solubility, suggesting that these synthases might be producing polysaccharides to strengthen or reinforce walls of cells that are undergoing secondary thickening. However, in this species transcript levels of *SvCslH1* are higher in a completely different set of tissues to *SvCslH2,* such that the two transcripts show a pronounced negative correlation, whilst *SvCslH2* transcripts are one of only a few that show a strong positive correlation with another member of the *CslF, CslH* or *CslJ* family (Additional file 1: Figure S4). Thus, the influence of different *CslH* genes on (1,3;1,4)-β-glucan properties is not clear. It has been observed previously that *CslF* transcript levels do not appear to be regulated in a coordinated pattern [45], in contrast to the *CesA*s where transcript co-expression is observed [43]. This may indicate the absence of a complex containing more than one CSLF, CSLH or CSLJ protein from the same family. In *Setaria* only the transcript patterns of *SvCslF4* and *SvCslH2* are highly correlated. *SvCslF4* could be a relatively important member of the *CslF* family in *Setaria*, given that transcript levels of this gene were second only to *SvCslF6* in a number of the more mature tissues, such as early stem internodes and mature roots (Additional file 1: Figure S2).

A specific role for *CslJ* genes in (1,3;1,4)-β-glucan synthesis has not yet been demonstrated unequivocally. The presence of *CslJ* genes across the Poales is patchy and these genes are absent from a number of important cereals, including rice [72]. *Setaria* is the only cereal so far examined that has more than a single *CslJ* gene. Transcripts of *SvCslJ2* were higher than *SvCslJ1* in all tissues tested and peaked in the mature root and the almost fully emerged inflorescence (Additional file 1: Figure S5). Our unpublished data (RA Ermawar, RA Burton and NJ Shirley) indicate that the sorghum *CslJ* is also found at high levels in root tissue suggesting that some cereal roots might require a particular cell wall structure conferred by the action of *CslJ*.

### The nature of *Setaria* grain

A previous report on the chemical composition of *S. viridis* grain suggested that it contains 11 % fibre [48] but no information regarding the constituent polysaccharides was provided. Here the amount of (1,3;1,4)-β-glucan in *S. viridis* has been determined with very little in the young grain (0.013 % w/w) decreasing even further to 0.003 % (w/w) at maturity (Table 1). Thus, the maximum amount of (1,3;1,4)-β-glucan is less than that found in other C_4_ grain, such as maize (0.1 % w/w, [73]) and sorghum (0.1–0.2 % w/w, [61]) and is considerably less than in the C_3_ cereals barley (4–10 % w/w) and oats (6–8 % w/w) [74]. In fact, *Setaria* has the lowest amount of (1,3;1,4)-β-glucan so far reported being even lower than rice grain at 0.006 % (w/w) [75].

Fluorescent immunolabelling in the very early stages of *Setaria* grain development, just a few days after pollination, indicated that the (1,3;1,4)-β-glucan is predominantly located in the outer layers of the caryopsis in the maternally-derived pericarp tissues (Fig. 9g) and is present in specific areas of the embryo, the coleoptile and the coleorhiza (Fig. 9j). It persists in the pericarp in the intermediate stages of grain development but the amount decreases in the embryo and is negligible in walls of the starchy endosperm, or aleurone cells of mature grain (Fig. 9k). By the time the grain is mature almost no (1,3;1,4)-β-glucan can be observed with sensitive antibody-based immunocytochemical methods, consistent with the results of the biochemical analyses.

In developing sorghum grain, (1,3;1,4)-β-glucan is deposited more in the outer maternal layers, such as the pericarp and testa, than in the endosperm tissues [61]. Sorghum grain very often displays dual morphology in the starchy endosperm where a floury centre is surrounded by a more glassy outer layer [76]. Although this morphological feature is not evident in *Setaria* grain the accumulation of (1,3;1,4)-β-glucan in both C_4_ grains is very similar. In contrast, the appearance of (1,3;1,4)-β-glucan in developing barley grain, a C_3_ species, is detected as early at 3 or 4 DAP in the maternal tissues, in the walls of starchy endosperm cells from 6 DAP onward [77] and continues to increase as the grain matures. Here, an examination of the transcript levels of the *CslF, H* and *J* genes across a *Setaria* developing grain series reveals that minimal amounts of these transcripts can be detected, except for *SvCslF4* and *SvCslF6* in the very early stages at 2 to 4 DAP (Fig. 3, Additional file 1: Figure S2). This is similar to the *CslF6* transcript in wheat, which peaked before 10 DAP and continuously decreased throughout maturity [56]. In all other cereals significant amounts of *CslF6* or *CslH* transcripts are observed, where for barley [78], rice [79] *Brachypodium distachyon* [80] and other C_4_ grain such as sorghum [61], the transcript peaked in the mid stages and much larger amounts of (1,3;1,4)-β-glucan are present. Also absent from the *Setaria* grain transcriptome is *CslF9.* In barley, the *HvCslF9* transcripts peak during endosperm cellularisation in early grain development, whilst maximal amounts of *HvCslF6* are found later at 18–20 DAP*.* Hence *Setaria* is somewhat unusual amongst the Poales in terms of its transcript profile in developing grain and until data from other millet species such as *Pennisetum* (pearl), *Panicum* (proso) and *Eleusine* (finger) are available it will not be clear whether *Setaria* is an outlier in this Order.

The DP3:DP4 ratio of the small amount of (1,3;1,4)-β-glucan present in *Setaria* grain is lower (2.5:1 to 2.8:1, Table 1) than that found in sorghum [2.6 to 3.0:1, 61] or barley grain [2.8 to 3.3:1, 69]. Since ratio is linked to solubility this suggests that *Setaria* grain (1,3;1,4)-β-glucan is likely to be more soluble than the (1,3;1,4)-β-glucan found in sorghum or barley grain.

## Conclusion

The genes in the *cellulose synthase* superfamily have been defined in *Setaria* and the analyses reveal subtle differences in numbers and chromosomal distributions when compared with other cereal genomes. Transcript analyses of Poales-specific genes in the *CslF*, *CslH* and *CslJ* families indicate that the *CslF6* transcript dominates and that the abundance and distribution of (1,3;1,4)-β-glucans in *Setaria* vegetative tissues are very similar to those described for the other C_4_ grasses such as maize and sorghum [59–61]. These features indicate that *Setaria* it is a suitable model for these and other grasses in the context of manipulating this valuable polysaccharide [37, 70]. However, *Setaria* grain contains only a very small amount of (1,3;1,4)-β-glucan, and the *CslF6* transcript, and so may be less useful in a human health context than other cereal grains [26, 69].

## Methods

### Bioinformatic analysis of *Csl* genes in *Setaria*

The *CesA* and *Csl* sequences of *Setaria italica*, sorghum (*Sorghum bicolor*), barley (*Hordeum vulgare*), rice (*Oryza sativa*) and *Brachypodium* (*Brachypodium distachyon*) were obtained from the public databases Phytozome 9.0 [9], NCBI GenBank [81], RGAP [82] and GRAMENE [83]. The hmmalign program in the HMMER package [84] was used to assign the retrieved full length protein sequences to the *cellulose synthase* pfam HMM (PF03552). The full length protein alignment was back-translated to codons using pal2nal [85] and residue assignments with a posterior probability of <0.6 were manually removed. The Bayesian Markov Chain Monte Carlo (MCMC) package BEAST v1.8.0 [86] was used to reconstruct the phylogenetic tree of *Setaria*, sorghum, barley and rice *Csl* and *CesA* genes. The final codon alignment was partitioned into the three separate codon positions, and each partition was unlinked, that is, substitution model parameters, rate heterogeneity model and base frequency were free to vary across partitions. Two analyses, replicated once, were reconstructed; one with a relaxed-clock (log-normal distribution of nucleotide rate variation), and one with a strict clock prior. The GTR + IG substitution model, as selected by jModelTest, and a Yule tree prior were used. Convergence was monitored in TRACER v1.5 [87] by assessing the Effective Sample Size (ESS) and likelihood of the estimated parameters. Each analysis was run for at least 75,000,000 states, or until stationarity, logging every 1000 states. *CesA, CslD,* and *CslE* clades on the tree were collapsed using nw_utils [88].

### Plant material

The *S. viridis* A10 accession was grown in a greenhouse under a day/night temperature regime of 28 °C/15 °C (The Plant Accelerator, Waite Campus, University of Adelaide, South Australia). Plants were grown in a soil mix composed of coco peat (75 %) and sand (25 %) supplemented with 2.5 g L^−1^ agricultural lime and Osmocote® Exact® Mini (Scotts Australia Pty Ltd, NSW, Australia), 1.875 g L^−1^ iron sulphate and calcium nitrate, 1 g L^−1^ hydrated lime, 0.75 g L^−1^ dolomite lime, gypsum, superphosphate and MicroPlus (Langley Australia Pty Ltd, WA, Australia) and 0.125 g L^−1^ iron chelate. Soil pH ranged from 6–6.5. *S. viridis* plant development was defined based on growth stages of perennial forage grasses [89]. Tissues such as root, leaves, stem internodes and inflorescence were collected at various stages from vegetative-leaf (Additional file 1: Figure S11A) through to the reproductive-floral development stage (Additional file 1: Figure S11B). The flag leaf was harvested at three different stages: vegetative-leaf, inflorescence emergence (IE) and at anthesis. The 2^nd^ to 4th leaves were harvested from tillers at IE, while the 5^th^ and 6^th^ leaves were harvested from the plant at anthesis. Roots were harvested at IE and at anthesis. Stem internodes were harvested at anthesis. Whole grains were collected throughout development and at the ripening stages, from 2 to 24 DAP (Fig. 3). Glumes were removed manually using tweezers (Dumont, no.3, Kirwan, Queensland). Grain images (Fig. 3) were taken using the Stemi 2000 Stereomicroscope (Zeiss, Germany).

### Total RNA isolation and cDNA synthesis

*S. viridis* vegetative tissues and developing grain were snap frozen in liquid nitrogen immediately after harvest. Total RNA extraction and cDNA synthesis methods followed Burton *et al.* [38].

### Q-PCR of the *S. viridis CslF*, *CslH* and *CslJ* genes

Real-time quantitative PCR (Q-PCR) of the *S. viridis* genes was performed as described by Burton *et al.* [45]. Primer design for the *Cellulose synthase-like* genes in *S. viridis* (*SvCsl*) and the control genes was based on *S. italica* sequences [Phytozome 9.0, 9] and tested on a cDNA sample generated from RNA from various tissues. The normalised expression values for the *SvCsl* genes were subjected to a correlation analysis and groups of genes sharing Pearson correlation coefficients (R^2^) greater than or equal to 0.90 were ascribed as ‘coexpressed’. The PCR primers and PCR product sizes in base pairs together with optimal acquisition temperatures are listed in Additional file 2: Table S2.

### (1,3;1,4)-β-Glucan quantitative assay

Samples of each *S. viridis* vegetative tissue were harvested from five individual plants. Vegetative tissues were freeze-dried, while mature grain samples were oven-dried at 37 °C for two days. All samples were ground using a Retsch mill (Type MM 300) for 1 min at a frequency of 30 beats/second. The ground samples were weighed (15–20 mg) and assayed in four technical replicates. The samples were pre-treated with a series of 70–100 % ethanol washes before measurement using a small scale version of the Megazyme assay [90] according to Ermawar *et al.* [61]. The amount of glucose released from the (1,3;1,4)-β-glucan in the vegetative samples was quantified based on the colorimetric assay using GOPOD, Megazyme International Ireland Ltd., according to Ermawar *et al.* [61]. Due to the low level of (1,3;1,4)-β-glucan in the grain, the amount of glucose released from the lichenase digestion was quantified using high pH anion exchange chromatography with pulsed amperometric detection (HPAEC-PAD) on a Dionex ICS-5000 column against a glucose standard.

### Structural analysis of *S. viridis* (1,3;1,4)-β-glucan

Lichenase-treated extracts of *S. viridis* vegetative tissues and grain samples were purified using solid phase extraction (SPE) cartridges packed with graphitized carbon (Varian Bond Elut Carbon 50 mg ml^−1^ columns) according to Ermawar *et al.* [61]. The ratio of DP3:DP4 oligosaccharides in the vegetative tissue extracts were analysed using either high-performance liquid chromatography (HPLC) [91] or HPAEC-PAD on a Dionex ICS-5000 chromatograph. The grain extracts were analysed using HPAEC-PAD following Ermawar *et al.* [61].

### Statistical analysis of (1,3;1,4)-β-glucan quantitative and structural data

GenStat 15^th^ Ed. SP2 was used for statistical analysis. All data were initially analysed by one-way ANOVA using a 5 % least significance difference. Data was analysed with a *t*-test to identify values with a significant difference (*P* < 0.05).

### Tissue fixation, embedding and sectioning of vegetative tissues and grain

*S. viridis* vegetative tissue and grain samples were fixed in paraformaldehyde/glutaraldehyde and embedded in LR-white as described in Burton et al. [38]. Sections for toluidine blue staining, fluorescence and TEM microscopy were prepared following Wilson et al. [77]. Individual vegetative tissue and grain samples in resin blocks were transversely sectioned at 1 μm on a Reichert-Jung Ultracut microtome for toluidine blue staining and fluorescence microscopy. Sections for TEM were cut transversely at a thickness of 80-100 nm.

### Fluorescence and TEM immunocytochemistry

Detection of (1,3;1,4)-β-glucan in *S. viridis* vegetative tissues and grain was carried out using fluorescence and TEM immunocytochemistry following Burton *et al.* [38] and Wilson *et al.* [77]. These methods rely on the use of the BG1 monoclonal antibody that is specific for (1,3;1,4)-β-glucans [92]. In the present study, dilutions of 1:50 and 1:500 of the anti-mouse primary antibody BG-1 (Biosupplies Australia, Parkville, Victoria Australia) were used for the fluorescence and TEM, immunocytochemistry. A 1:100 dilution of Alexa Fluor® 488 goat anti-mouse IgG (H + L) secondary antibody was used for the fluorescence detection, and a 1:30 dilution of an Aurion goat IgG/IgM anti-mouse secondary antibody conjugated to either 10 or 25 nm gold was used for the TEM. Fluorescent sections were counterstained with 0.001 % Calcofluor White MR2® (Sigma-Aldrich, St.Louis, MO). Three types of negative control of fluorescence micrographs from various tissues of *Setaria* were applied. The cell walls show no labelling when they are treated with no antibody, the primary antibody only or secondary antibody only. Fluorescence images were taken with an Axio Imager M2 Microscope (Zeiss, Germany). TEM images were taken as described in Wilson et al. [77]. Control transmission electron micrographs of *H. vulgare* leaf sections labelled with the antibody BG-1 was used as positive control, while the sections labelled with the secondary antibody only was used as negative control (Additional file 1: Figure S12).

### Availability of supporting data

All the supporting data are included as additional files.

## Additional files

Additional file 1: Figure S1. Bayesian phylogenetic tree of the *CesA, CslD, CslE*, *CslF*, *CslH* and *CslJ* genes in *Setaria italica*, *Sorghum bicolor*, *Oriza sativa*, *Brachypodium distachyon* and *Hordeum vulgare.* **Figure S2.** Transcript levels of (1,3;1,4)-β-glucan synthase genes from *S. viridis.* **Figure S3.** Comparison of the transcripts levels of *SvCslH* genes from *S. viridis*. **Figure S4.** Correlation of the *Cellulose synthase-like* transcript levels. **Figure S5.** Normalised transcript levels of *SvCslJ* from *S. viridis.* **Figure S6.** TEM micrographs of leaf immunogold labelled with BG-1. **Figure S7.** TEM micrographs of stem sections immunogold labelled with BG-1 **Figure S8.** TEM micrographs of root sections immunogold labelled with BG-1. **Figure S9.** Micrographs of developing grain transverse sections. **Figure S10.** TEM micrographs of grain immunogold labelled with BG-1. **Figure S11.** Phenotype of *Setaria viridis* in different growth stages. **Figure S12.** Control micrographs of TEM of *H. vulgare* leaf sections. (PPTX 17100 kb) Additional file 2: Table S1.
*Cellulose synthase* (*CesA*) and *Cellulose synthase-like* (*Csl*) gene families in *S. italica*. **Table S2.** Primer sequences for the amplification of transcripts of control genes and *Cellulose synthase-like* genes (*Csl*) *F*, *H* and *J* in *S. viridis*. (DOCX 19 kb)

## Abbreviations

BLAST: Basic local alignment search tool
bp: Base pairs
cDNA: Complementary DNA
*Ces*: *Cellulose synthase*
*Csl*: *Cellulose synthase-like*
DAP: Days after pollination
DP: Degree of polymerisation
GOPOD: Glucose Oxidase/Peroxidase
HPAEC-PAD: High pH anion exchange chromatography with pulsed amperometric detection
HPLC: High-performance liquid chromatography
NCBI: National Center for Biotechnology Information
PCR: Polymerase chain reaction
Q-PCR: Quantitative PCR
RGAP: Rice genome annotation project
TEM: Transmission electron microscopy
Tm: Temperature
w/w: Weight/weight
