# Supplementary material for: Genetics and physiology of cell wall polysaccharides in the model C4 grass, Setaria viridis spp
Source: BMC Plant Biol. 2015 Oct 2;15:236. doi: 10.1186/s12870-015-0624-0 (PMC4592572; doi:10.1186/s12870-015-0624-0)
Supplement: Additional file 1: Figure S1. — Bayesian phylogenetic tree of the CesA, CslD, CslE, CslF, CslH and CslJ genes in Setaria italica, Sorghum bicolor, Oriza sativa, Brachypodium distachyon and Hordeum vulgare. Figure S2. Transcript levels of (1,3;1,4)-β-glucan synthase genes from S. viridis. Figure S3. Comparison of the transcripts levels of SvCslH genes from S. viridis. Figure S4. Correlation of the Cellulose synthase-like transcript levels. Figure S5. Normalised transcript levels of SvCslJ from S. viridis. Figure S6. TEM micrographs of leaf immunogold labelled with BG-1. Figure S7. TEM micrographs of stem sections immunogold labelled with BG-1 Figure S8. TEM micrographs of root sections immunogold labelled with BG-1. Figure S9. Micrographs of developing grain transverse sections. Figure S10. TEM micrographs of grain immunogold labelled with BG-1. Figure S11. Phenotype of Setaria viridis in different growth stages. Figure S12. Control micrographs of TEM of H. vulgare leaf sections. (PPTX 17100 kb) [file 12870_2015_624_MOESM1_ESM.pptx]

## Slide 1
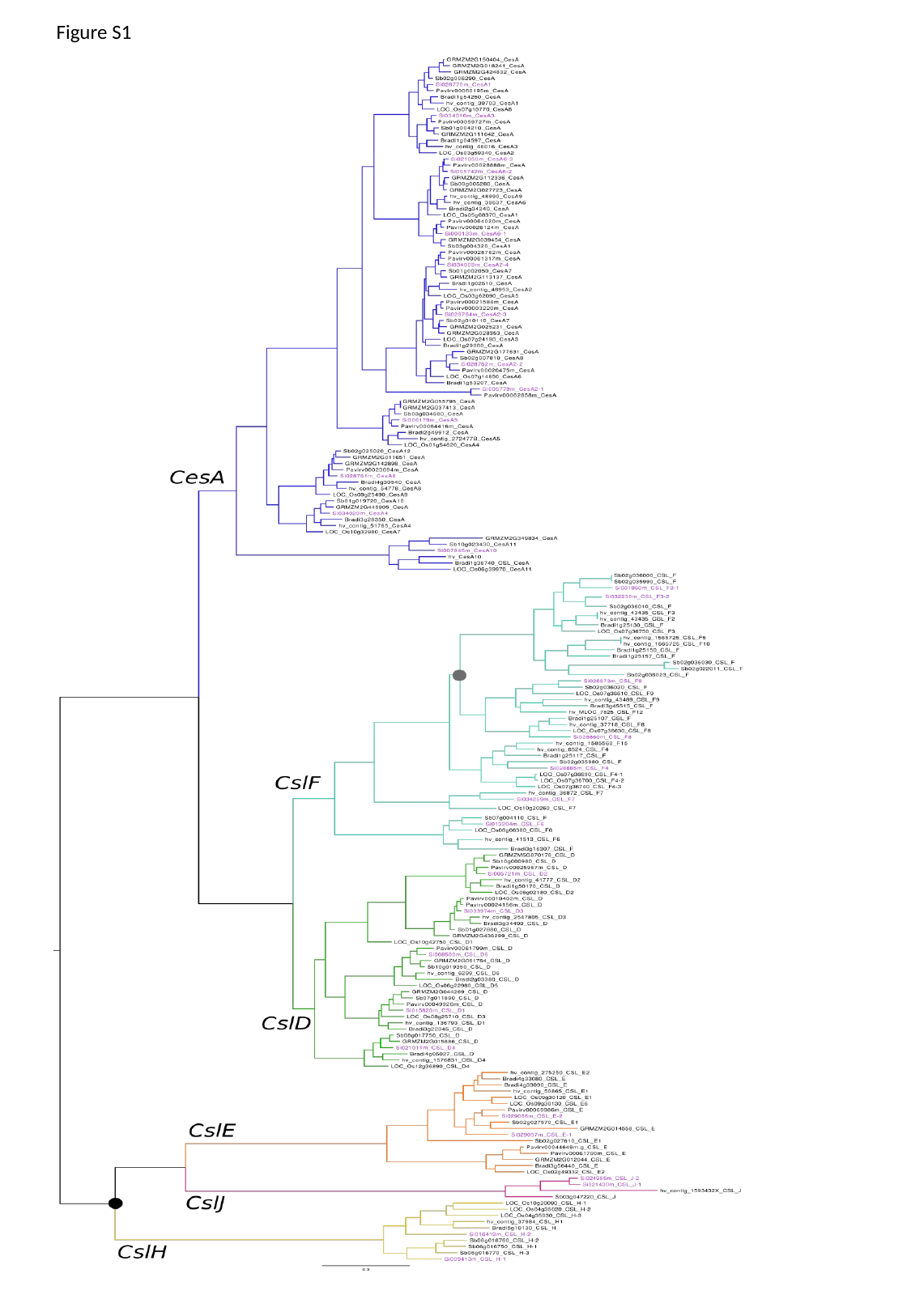

# Figure S1

## Slide 2
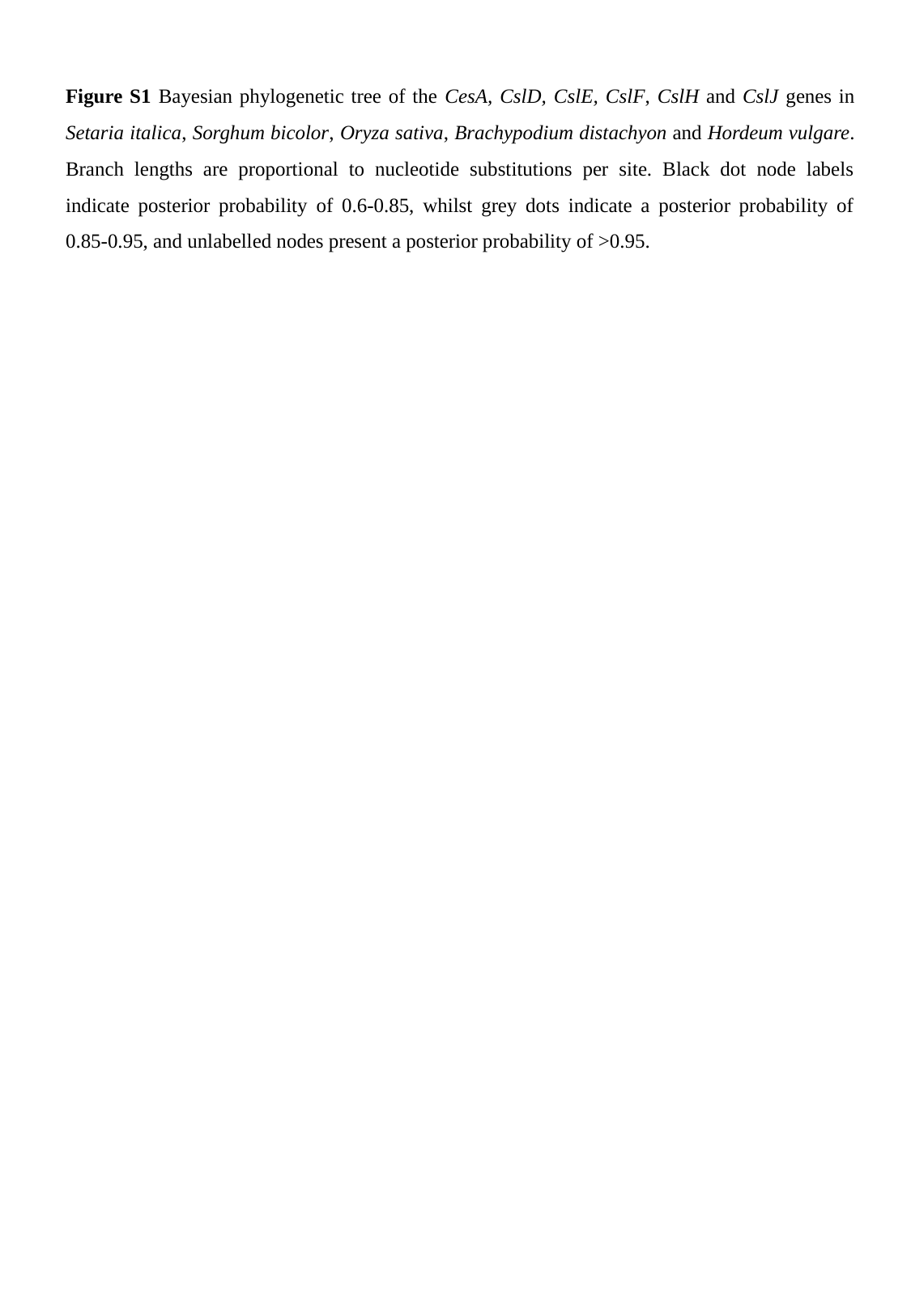

Figure S1 Bayesian phylogenetic tree of the CesA, CslD, CslE, CslF, CslH and CslJ genes in Setaria italica, Sorghum bicolor, Oryza sativa, Brachypodium distachyon and Hordeum vulgare. Branch lengths are proportional to nucleotide substitutions per site. Black dot node labels indicate posterior probability of 0.6-0.85, whilst grey dots indicate a posterior probability of 0.85-0.95, and unlabelled nodes present a posterior probability of >0.95.

## Slide 3
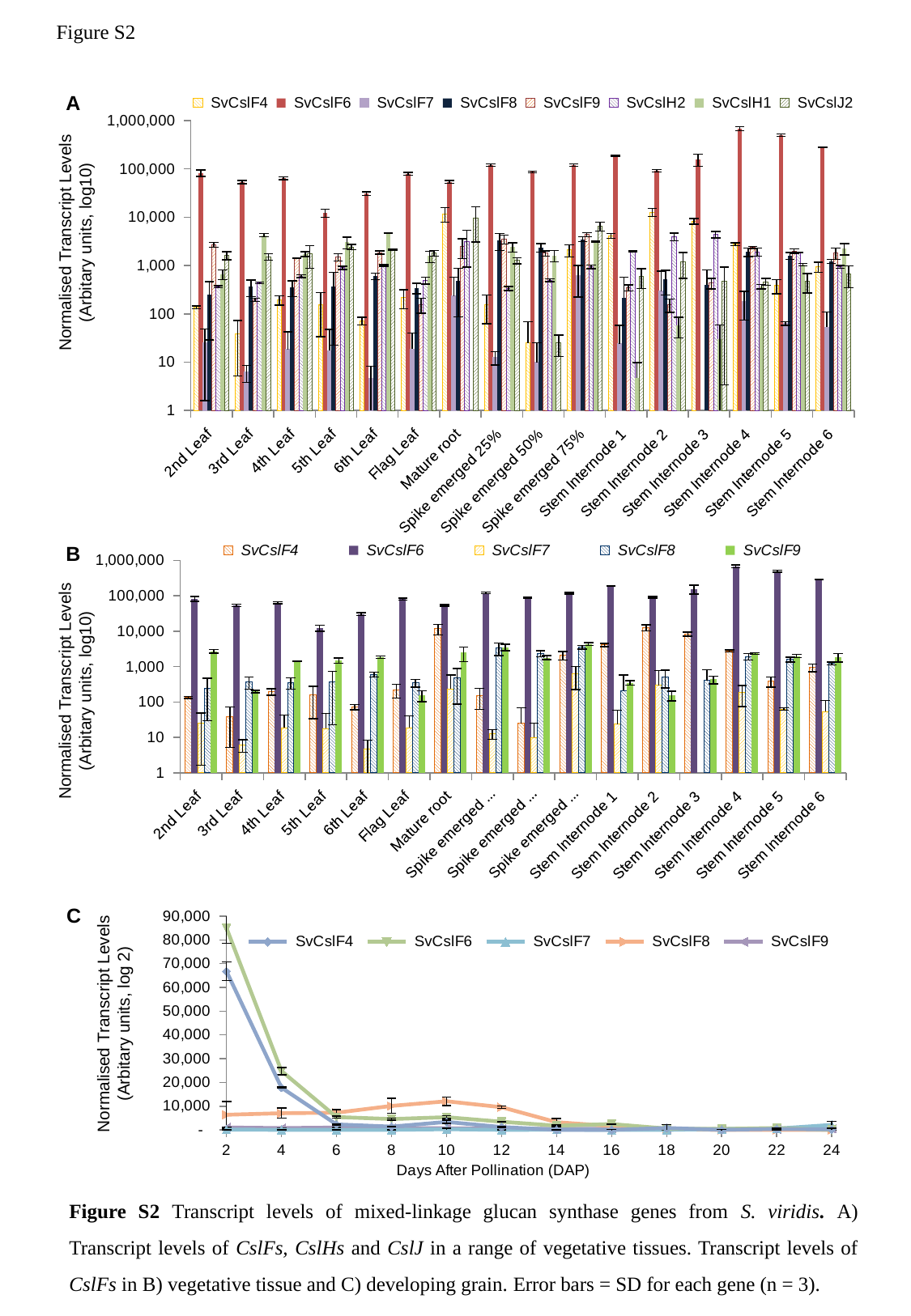

# Figure S2
a
### Chart
| Category | SvCslF4 | SvCslF6 | SvCslF7 | SvCslF8 | SvCslF9 | SvCslH2 | SvCslH1 | SvCslJ2 |
|---|---|---|---|---|---|---|---|---|
| 2nd Leaf | 135.9678679632291 | 82808.50822762305 | 25.21400613226406 | 249.25643943153332 | 2706.1682362699125 | 366.1780147792784 | 661.8541720961416 | 1625.859161888348 |
| 3rd Leaf | 39.1068886815431 | 53815.004613856276 | 6.175566591023601 | 365.8532481674862 | 197.34634558601093 | 439.08429454026 | 4256.460200512354 | 1525.0176667319895 |
| 4th Leaf | 194.14030516327944 | 64371.80067647418 | 18.498938432527137 | 355.61886210474535 | 1430.3593186794192 | 602.5529413334561 | 1744.3062951218508 | 1738.6749592215383 |
| 5th Leaf | 156.32986351620136 | 12334.56287832405 | 17.444284565691056 | 374.10198612967974 | 1503.1158534103793 | 905.6491070354606 | 3055.6571797568836 | 2436.385077674851 |
| 6th Leaf | 71.90413652113601 | 30694.599969992734 | 4.553928646338615 | 603.3746779385543 | 1855.4367566648034 | 1000.4572229060217 | 4679.134854211239 | 2124.9282136096913 |
| Flag Leaf | 222.90638436598977 | 79976.55934541539 | 18.626860322913103 | 345.7756967582957 | 156.00261011607384 | 492.7516071739841 | 1571.7077125377077 | 1807.9948517536807 |
| Mature root | 11816.055671623479 | 53912.17693719537 | 234.0140608157878 | 479.3205370711232 | 2480.0815629329177 | 3171.893788382626 | 0.0 | 9707.834563353084 |
| Spike emerged 25% | 153.41221110680016 | 120923.8880004115 | 12.636156816878477 | 3360.0405093840395 | 3515.331237361535 | 336.37975431254307 | 2439.5672427432382 | 1271.7559214608616 |
| Spike emerged 50% | 24.916963073168798 | 86824.93761153334 | 9.871863465179258 | 2355.2462714400035 | 1796.3943853703602 | 497.15273941227275 | 1627.9082541136947 | 24.845771750102603 |
| Spike emerged 75% | 2106.84983077439 | 119345.01928379251 | 614.7135562763115 | 3520.0439433085626 | 4356.917135712804 | 942.9921642245732 | 3140.4313611870507 | 6505.179248173177 |
| Stem Internode 1 | 4119.035150496572 | 188246.93797408682 | 23.85561597493342 | 211.6880598458256 | 349.7293995704636 | 1979.9495107957464 | 4.718571264045938 | 601.7566176736232 |
| Stem Internode 2 | 12757.463691818968 | 91655.5643878256 | 295.83691289232627 | 520.9297674159411 | 154.82358849294863 | 3973.8054379856812 | 58.213669273348685 | 1202.463203961901 |
| Stem Internode 3 | 8328.87479990984 | 157937.6765390903 | 0.0 | 403.9504277956272 | 434.4341095632972 | 4375.435561552636 | 29.628583954879264 | 471.74746866689327 |
| Stem Internode 4 | 2791.24505150599 | 685079.7292566405 | 183.02753554424206 | 1930.9555268830134 | 2355.4652049720808 | 1934.7122496979612 | 368.9101804278442 | 463.57959536452114 |
| Stem Internode 5 | 386.55392036741773 | 498622.16448734596 | 63.20192589992417 | 1601.6433385800829 | 1997.5551750830248 | 1889.5792196731315 | 1050.9659659896274 | 475.0942038035302 |
| Stem Internode 6 | 949.3782052610468 | 284880.00677961734 | 53.259451863131694 | 1231.0862241118714 | 1834.4293825955272 | 940.5055117539341 | 2238.13693716915 | 664.1211090073771 |Normalised Transcript Levels
(Arbitary units, log10)
### Chart
| Category | SvCslF4 | SvCslF6 | SvCslF7 | SvCslF8 | SvCslF9 |
|---|---|---|---|---|---|
| 2nd Leaf | 135.9678679632291 | 82808.50822762305 | 25.21400613226406 | 249.25643943153332 | 2706.1682362699125 |
| 3rd Leaf | 39.1068886815431 | 53815.004613856276 | 6.175566591023601 | 365.8532481674862 | 197.34634558601093 |
| 4th Leaf | 194.14030516327944 | 64371.80067647418 | 18.498938432527137 | 355.61886210474535 | 1430.3593186794192 |
| 5th Leaf | 156.32986351620136 | 12334.56287832405 | 17.444284565691056 | 374.10198612967974 | 1503.1158534103793 |
| 6th Leaf | 71.90413652113601 | 30694.599969992734 | 4.553928646338615 | 603.3746779385543 | 1855.4367566648034 |
| Flag Leaf | 222.90638436598977 | 79976.55934541539 | 18.626860322913103 | 345.7756967582957 | 156.00261011607384 |
| Mature root | 11816.055671623479 | 53912.17693719537 | 234.0140608157878 | 479.3205370711232 | 2480.0815629329177 |
| Spike emerged 25% | 153.41221110680016 | 120923.8880004115 | 12.636156816878477 | 3360.0405093840395 | 3515.331237361535 |
| Spike emerged 50% | 24.916963073168798 | 86824.93761153334 | 9.871863465179258 | 2355.2462714400035 | 1796.3943853703602 |
| Spike emerged 75% | 2106.84983077439 | 119345.01928379251 | 614.7135562763115 | 3520.0439433085626 | 4356.917135712804 |
| Stem Internode 1 | 4119.035150496572 | 188246.93797408682 | 23.85561597493342 | 211.6880598458256 | 349.7293995704636 |
| Stem Internode 2 | 12757.463691818968 | 91655.5643878256 | 295.83691289232627 | 520.9297674159411 | 154.82358849294863 |
| Stem Internode 3 | 8328.87479990984 | 157937.6765390903 | 0.0 | 403.9504277956272 | 434.4341095632972 |
| Stem Internode 4 | 2791.24505150599 | 685079.7292566405 | 183.02753554424206 | 1930.9555268830134 | 2355.4652049720808 |
| Stem Internode 5 | 386.55392036741773 | 498622.16448734596 | 63.20192589992417 | 1601.6433385800829 | 1997.5551750830248 |
| Stem Internode 6 | 949.3782052610468 | 284880.00677961734 | 53.259451863131694 | 1231.0862241118714 | 1834.4293825955272 |b
Normalised Transcript Levels
(Arbitary units, log10)
c
### Chart
| Category | SvCslF4 | SvCslF6 | SvCslF7 | SvCslF8 | SvCslF9 |
|---|---|---|---|---|---|Normalised Transcript Levels
(Arbitary units, log 2)
Figure S2 Transcript levels of mixed-linkage glucan synthase genes from S. viridis. A) Transcript levels of CslFs, CslHs and CslJ in a range of vegetative tissues. Transcript levels of CslFs in B) vegetative tissue and C) developing grain. Error bars = SD for each gene (n = 3).

## Slide 4
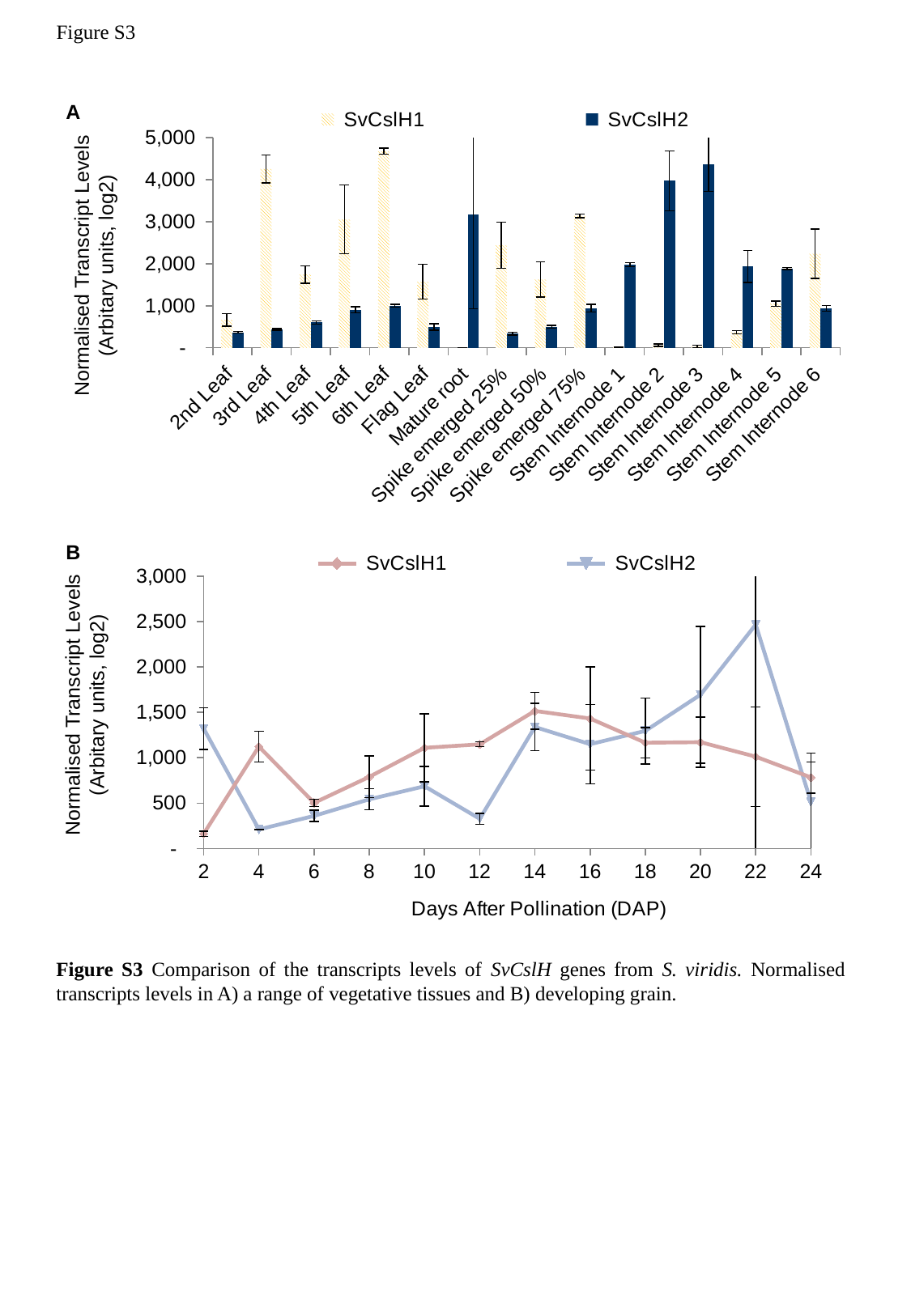

# Figure S3
A
### Chart
| Category | SvCslH1 | SvCslH2 |
|---|---|---|
| 2nd Leaf | 661.8541720961416 | 366.1780147792784 |
| 3rd Leaf | 4256.460200512354 | 439.08429454026 |
| 4th Leaf | 1744.3062951218508 | 602.5529413334561 |
| 5th Leaf | 3055.6571797568836 | 905.6491070354606 |
| 6th Leaf | 4679.134854211239 | 1000.4572229060217 |
| Flag Leaf | 1571.7077125377077 | 492.7516071739841 |
| Mature root | 0.0 | 3171.893788382626 |
| Spike emerged 25% | 2439.5672427432382 | 336.37975431254307 |
| Spike emerged 50% | 1627.9082541136947 | 497.15273941227275 |
| Spike emerged 75% | 3140.4313611870507 | 942.9921642245732 |
| Stem Internode 1 | 4.718571264045938 | 1979.9495107957464 |
| Stem Internode 2 | 58.213669273348685 | 3973.8054379856812 |
| Stem Internode 3 | 29.628583954879264 | 4375.435561552636 |
| Stem Internode 4 | 368.9101804278442 | 1934.7122496979612 |
| Stem Internode 5 | 1050.9659659896274 | 1889.5792196731315 |
| Stem Internode 6 | 2238.13693716915 | 940.5055117539341 |Normalised Transcript Levels
(Arbitary units, log2)
B
### Chart
| Category | SvCslH1 | SvCslH2 |
|---|---|---|Normalised Transcript Levels
(Arbitary units, log2)
Figure S3 Comparison of the transcripts levels of SvCslH genes from S. viridis. Normalised transcripts levels in A) a range of vegetative tissues and B) developing grain.

## Slide 5
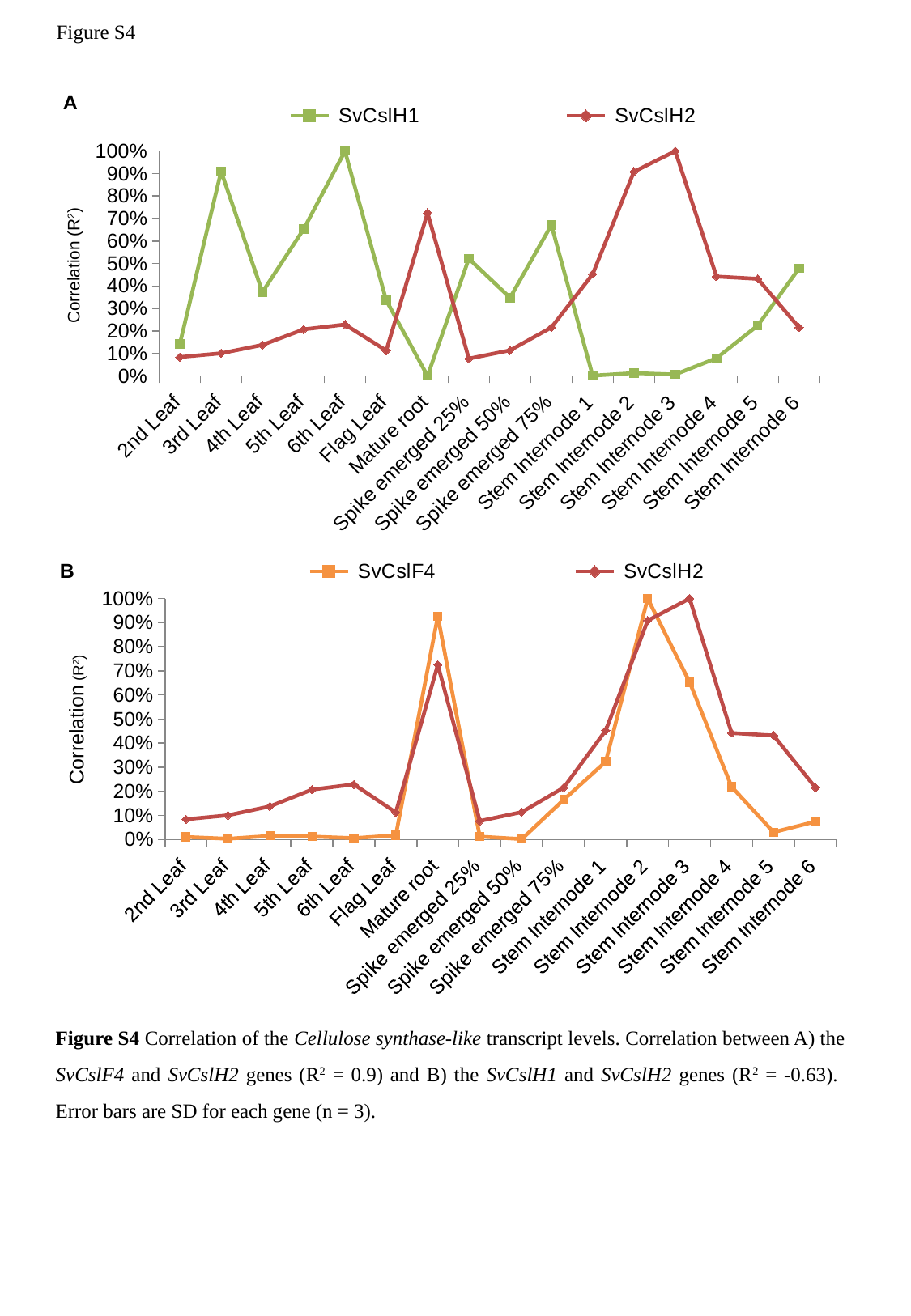

Figure S4
A
### Chart
| Category | SvCslH1 | SvCslH2 |
|---|---|---|
| 2nd Leaf | 0.14144797974789514 | 0.08368950008015638 |
| 3rd Leaf | 0.9096682043009562 | 0.100352133716363 |
| 4th Leaf | 0.37278393324184034 | 0.13771267633973303 |
| 5th Leaf | 0.6530389217157913 | 0.2069849033987575 |
| 6th Leaf | 1.0 | 0.22865317265717122 |
| Flag Leaf | 0.3358970753157851 | 0.11261772690788521 |
| Mature root | 0.0 | 0.7249321224735557 |
| Spike emerged 25% | 0.5213714327014145 | 0.07687914713413761 |
| Spike emerged 50% | 0.3479079583800777 | 0.11362359984930431 |
| Spike emerged 75% | 0.6711564122501514 | 0.21551960963857728 |
| Stem Internode 1 | 0.0010084281413259987 | 0.452514837195581 |
| Stem Internode 2 | 0.012441118088519327 | 0.9082079674316047 |
| Stem Internode 3 | 0.006332064554244131 | 1.0 |
| Stem Internode 4 | 0.07884153629293748 | 0.4421759211125082 |
| Stem Internode 5 | 0.22460689822687074 | 0.4318608269030499 |
| Stem Internode 6 | 0.47832281113993075 | 0.21495128851130724 |Correlation (R2)
### Chart
| Category | SvCslF4 | SvCslH2 |
|---|---|---|
| 2nd Leaf | 0.010657907500095163 | 0.08368950008015638 |
| 3rd Leaf | 0.0030654125009676753 | 0.100352133716363 |
| 4th Leaf | 0.015217782300080274 | 0.13771267633973303 |
| 5th Leaf | 0.012253992430834953 | 0.2069849033987575 |
| 6th Leaf | 0.005636240733904363 | 0.22865317265717122 |
| Flag Leaf | 0.01747262541761603 | 0.11261772690788521 |
| Mature root | 0.9262072741936008 | 0.7249321224735557 |
| Spike emerged 25% | 0.012025290826826296 | 0.07687914713413761 |
| Spike emerged 50% | 0.0019531282765199954 | 0.11362359984930431 |
| Spike emerged 75% | 0.16514644929975056 | 0.21551960963857728 |
| Stem Internode 1 | 0.32287257483146925 | 0.452514837195581 |
| Stem Internode 2 | 1.0 | 0.9082079674316047 |
| Stem Internode 3 | 0.652862904501224 | 1.0 |
| Stem Internode 4 | 0.2187931017429384 | 0.4421759211125082 |
| Stem Internode 5 | 0.030300217167406465 | 0.4318608269030499 |
| Stem Internode 6 | 0.07441747264151405 | 0.21495128851130724 |B
Correlation (R2)
Figure S4 Correlation of the Cellulose synthase-like transcript levels. Correlation between A) the SvCslF4 and SvCslH2 genes (R2 = 0.9) and B) the SvCslH1 and SvCslH2 genes (R2 = -0.63). Error bars are SD for each gene (n = 3).

## Slide 6
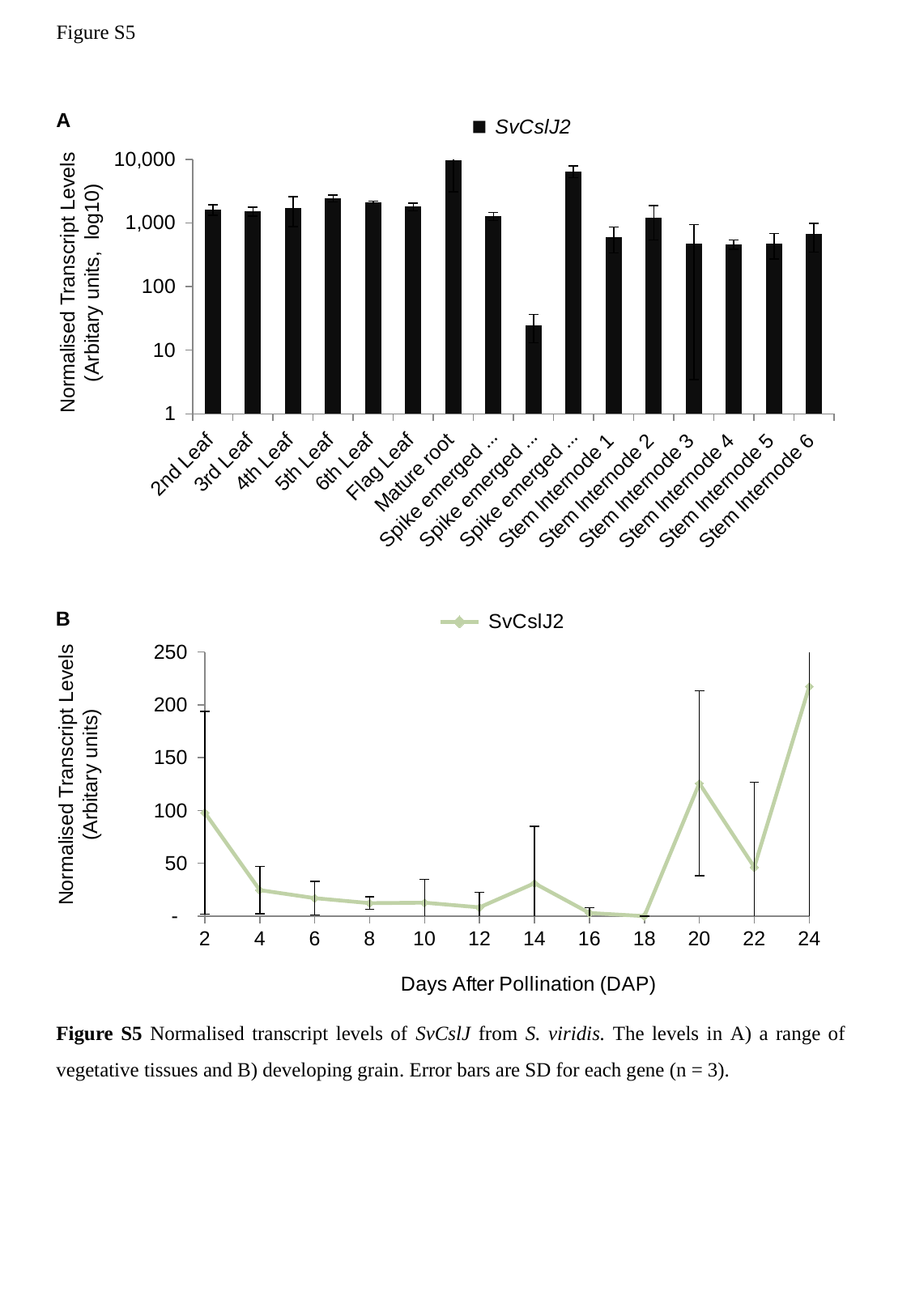

# Figure S5
A
### Chart
| Category | SvCslJ2 |
|---|---|
| 2nd Leaf | 1625.859161888348 |
| 3rd Leaf | 1525.0176667319895 |
| 4th Leaf | 1738.6749592215383 |
| 5th Leaf | 2436.385077674851 |
| 6th Leaf | 2124.9282136096913 |
| Flag Leaf | 1807.9948517536807 |
| Mature root | 9707.834563353084 |
| Spike emerged 25% | 1271.7559214608616 |
| Spike emerged 50% | 24.845771750102603 |
| Spike emerged 75% | 6505.179248173177 |
| Stem Internode 1 | 601.7566176736232 |
| Stem Internode 2 | 1202.463203961901 |
| Stem Internode 3 | 471.74746866689327 |
| Stem Internode 4 | 463.57959536452114 |
| Stem Internode 5 | 475.0942038035302 |
| Stem Internode 6 | 664.1211090073771 |Normalised Transcript Levels
(Arbitary units, log10)
B
### Chart
| Category | SvCslJ2 |
|---|---|Normalised Transcript Levels
(Arbitary units)
Figure S5 Normalised transcript levels of SvCslJ from S. viridis. The levels in A) a range of vegetative tissues and B) developing grain. Error bars are SD for each gene (n = 3).

## Slide 7
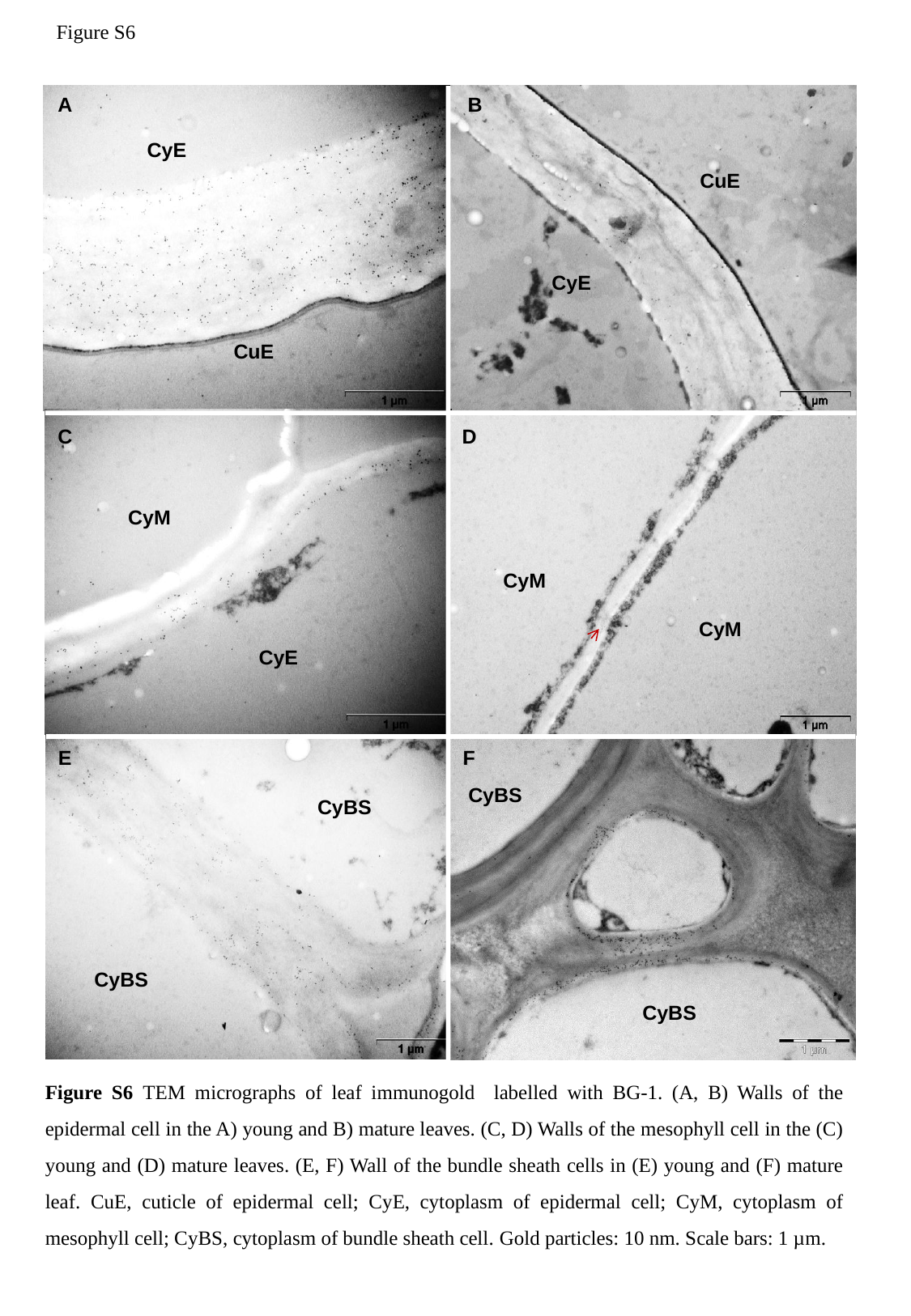

# Figure S6
A
B
CyE
CuE
CyE
CuE
C
D
CyM
CyM
CyM
CyE
F
E
CyBS
CyBS
CyBS
CyBS
Figure S6 TEM micrographs of leaf immunogold labelled with BG-1. (A, B) Walls of the epidermal cell in the A) young and B) mature leaves. (C, D) Walls of the mesophyll cell in the (C) young and (D) mature leaves. (E, F) Wall of the bundle sheath cells in (E) young and (F) mature leaf. CuE, cuticle of epidermal cell; CyE, cytoplasm of epidermal cell; CyM, cytoplasm of mesophyll cell; CyBS, cytoplasm of bundle sheath cell. Gold particles: 10 nm. Scale bars: 1 µm.

## Slide 8
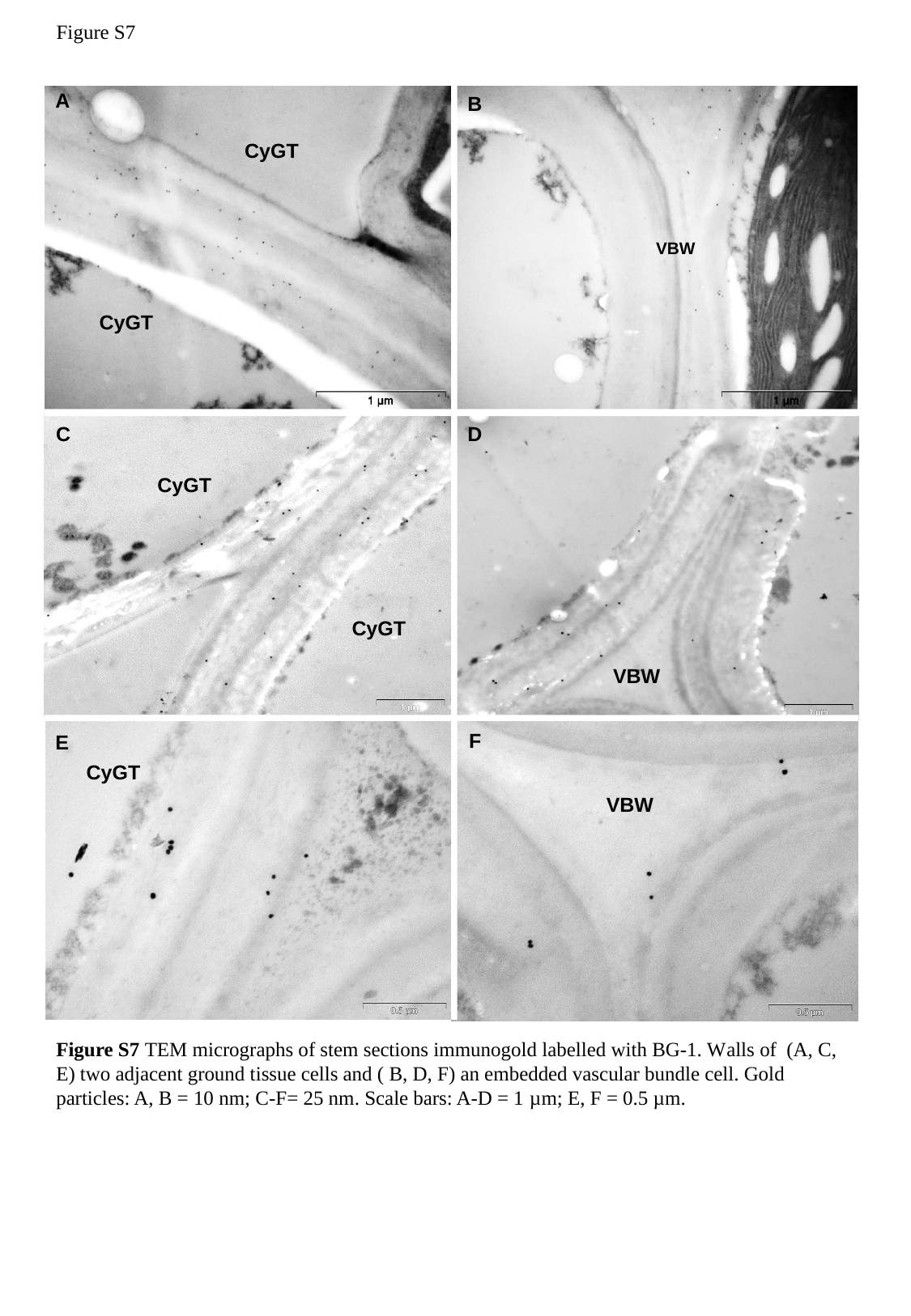

Figure S7
A
B
cygt
VBW
cygt
C
D
CyGT
CyGT
VBW
F
E
CyGT
VBW
Figure S7 TEM micrographs of stem sections immunogold labelled with BG-1. Walls of (A, C, E) two adjacent ground tissue cells and ( B, D, F) an embedded vascular bundle cell. Gold particles: A, B = 10 nm; C-F= 25 nm. Scale bars: A-D = 1 µm; E, F = 0.5 µm.

## Slide 9
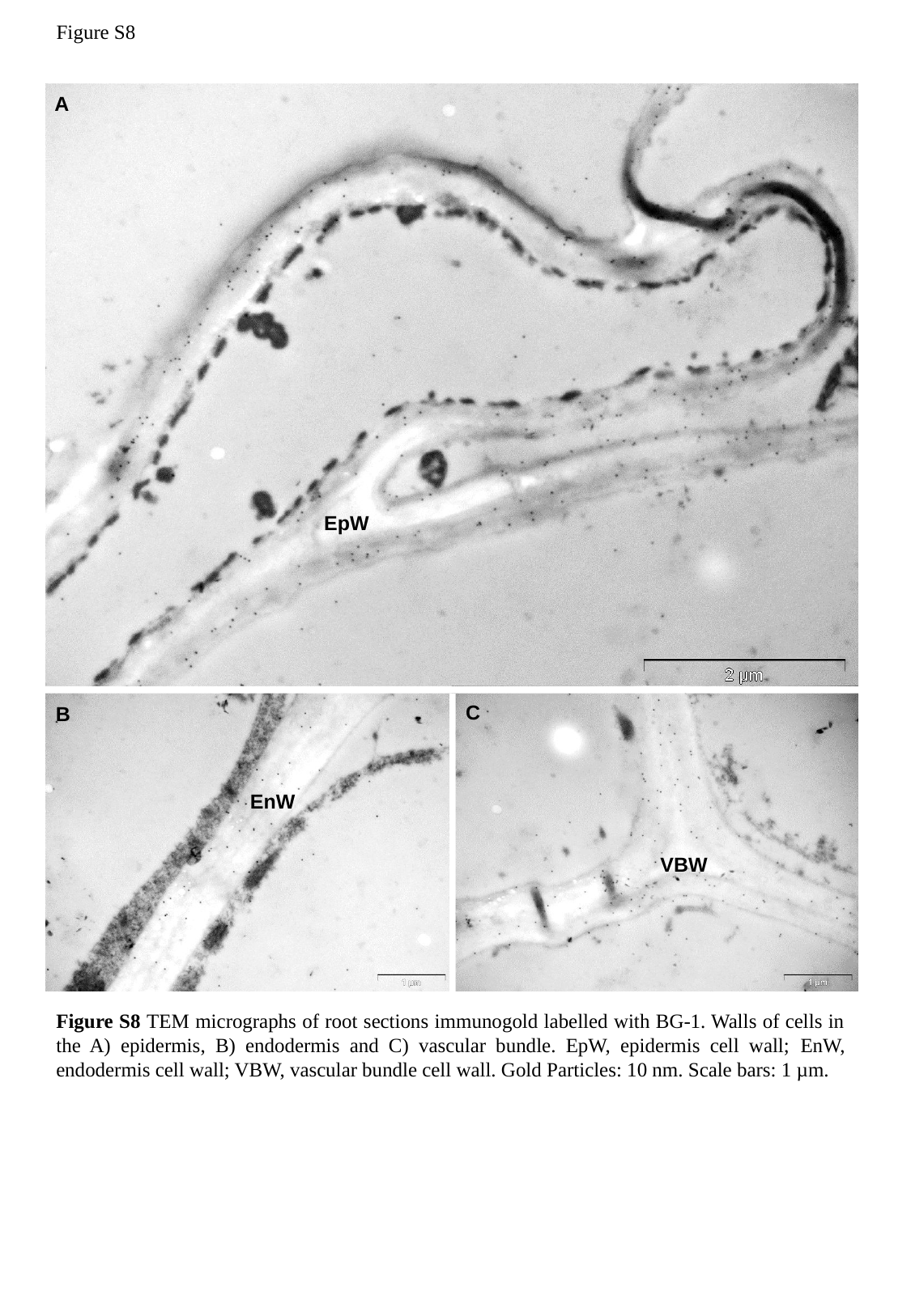

Figure S8
A
EpW
C
B
EnW
VBW
Figure S8 TEM micrographs of root sections immunogold labelled with BG-1. Walls of cells in the A) epidermis, B) endodermis and C) vascular bundle. EpW, epidermis cell wall; EnW, endodermis cell wall; VBW, vascular bundle cell wall. Gold Particles: 10 nm. Scale bars: 1 µm.

## Slide 10
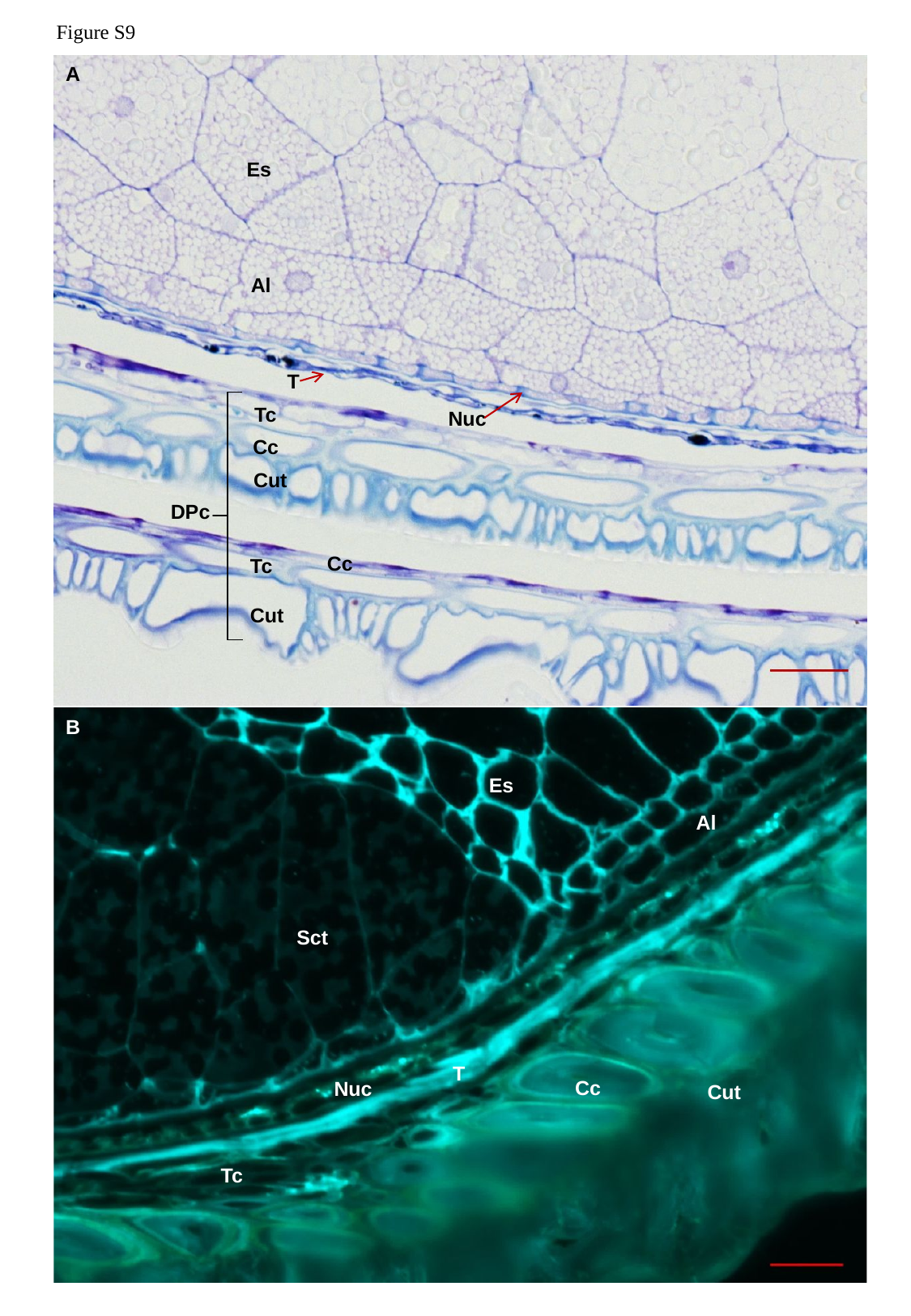

Figure S9
A
Es
Al
T
Tc
Nuc
Cc
Cut
DPc
Cc
Tc
Cut
b
Es
Al
Sct
T
Cc
Nuc
Cut
Tc

## Slide 11
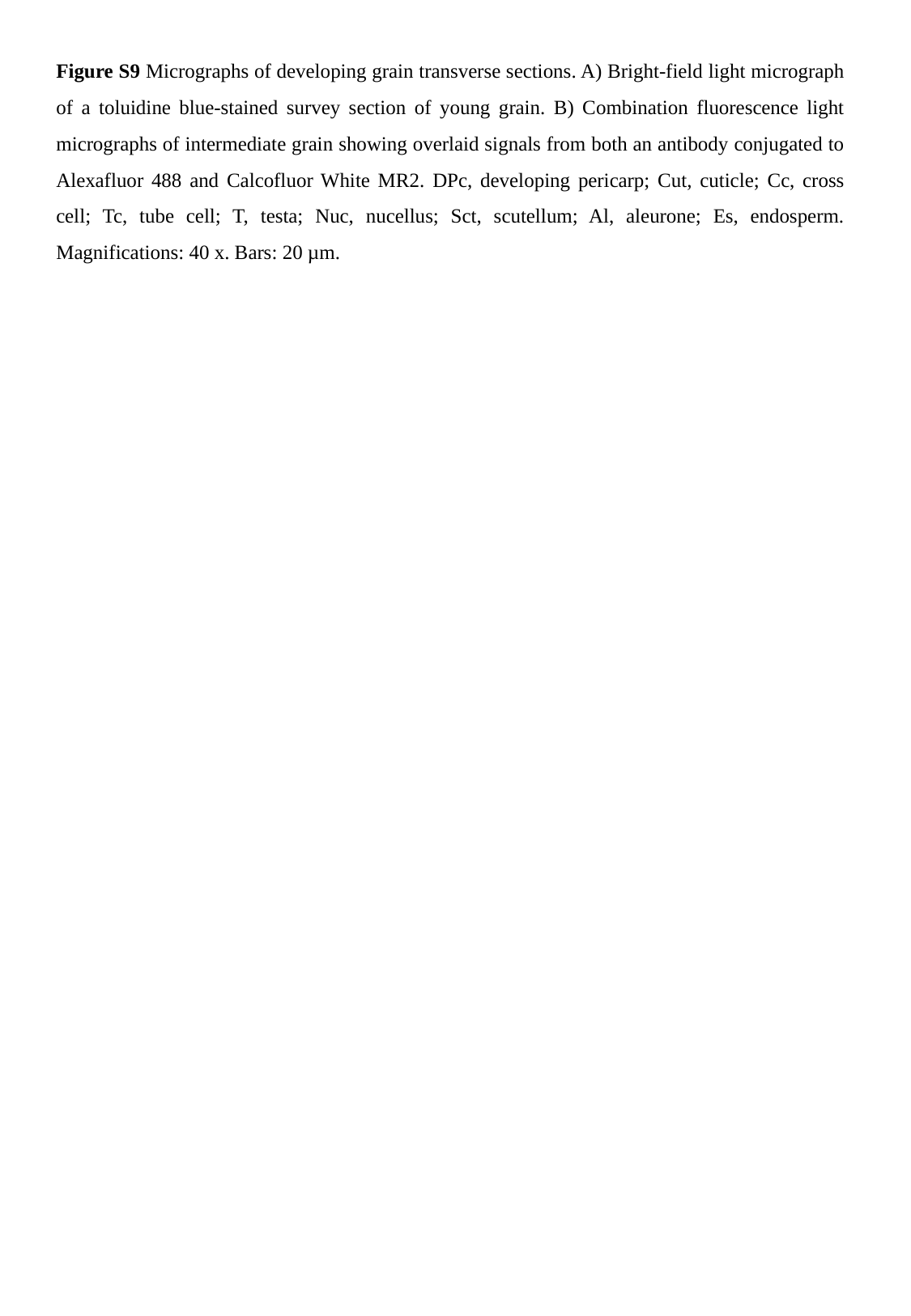

Figure S9 Micrographs of developing grain transverse sections. A) Bright-field light micrograph of a toluidine blue-stained survey section of young grain. B) Combination fluorescence light micrographs of intermediate grain showing overlaid signals from both an antibody conjugated to Alexafluor 488 and Calcofluor White MR2. DPc, developing pericarp; Cut, cuticle; Cc, cross cell; Tc, tube cell; T, testa; Nuc, nucellus; Sct, scutellum; Al, aleurone; Es, endosperm. Magnifications: 40 x. Bars: 20 µm.

## Slide 12
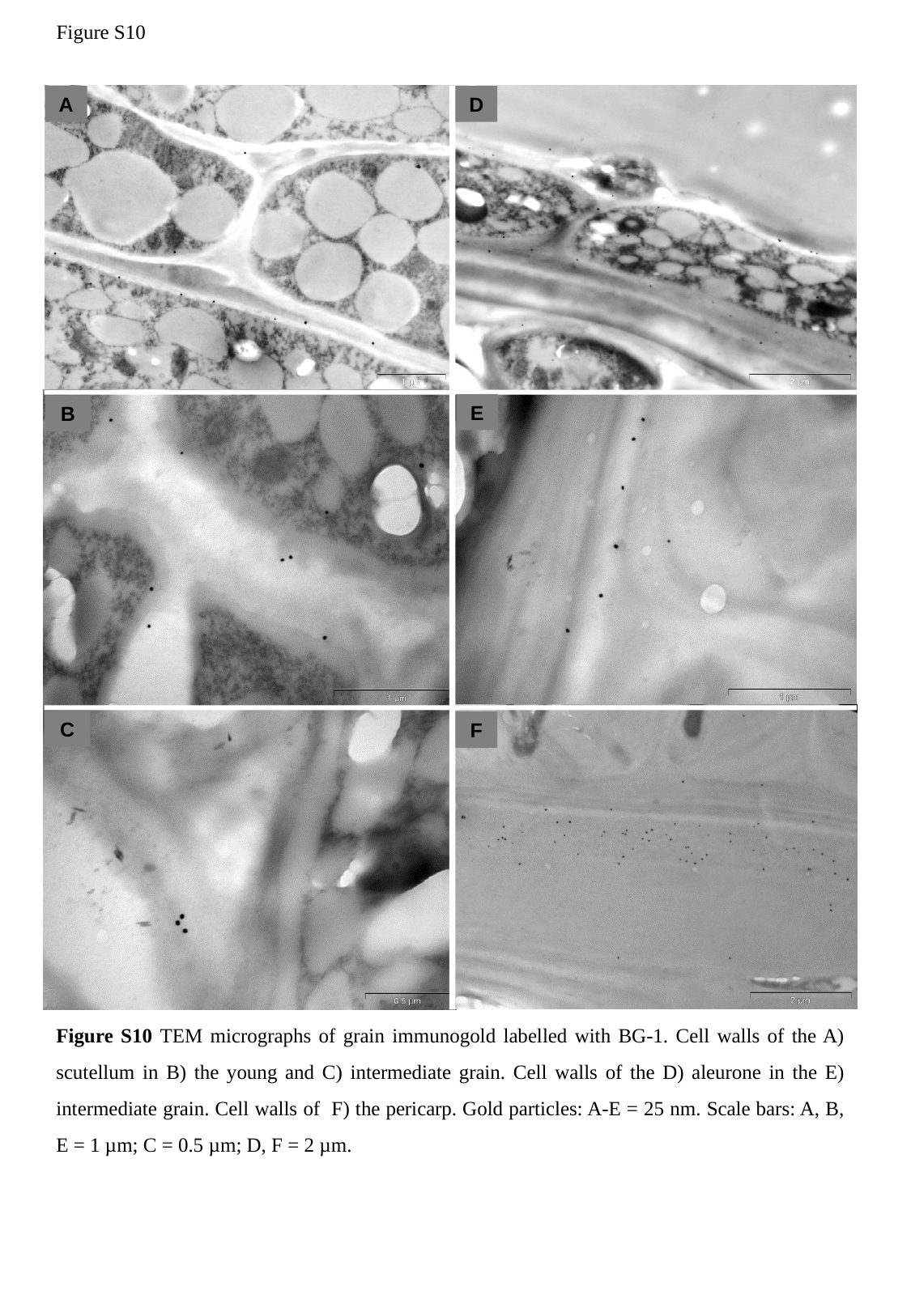

Figure S10
D
A
E
B
C
F
Figure S10 TEM micrographs of grain immunogold labelled with BG-1. Cell walls of the A) scutellum in B) the young and C) intermediate grain. Cell walls of the D) aleurone in the E) intermediate grain. Cell walls of F) the pericarp. Gold particles: A-E = 25 nm. Scale bars: A, B, E = 1 µm; C = 0.5 µm; D, F = 2 µm.

## Slide 13
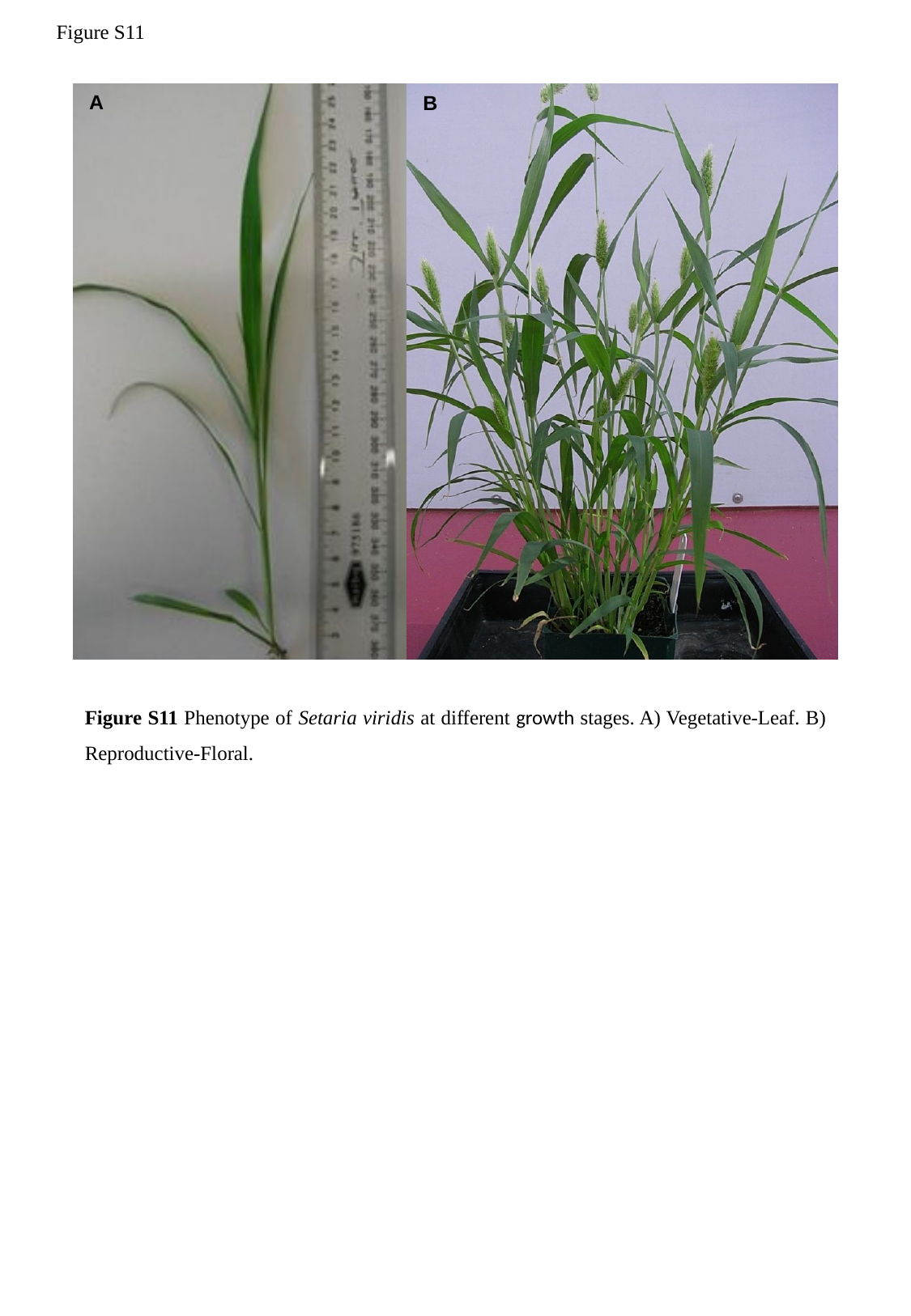

Figure S11
a
b
Figure S11 Phenotype of Setaria viridis at different growth stages. A) Vegetative-Leaf. B) Reproductive-Floral.

## Slide 14
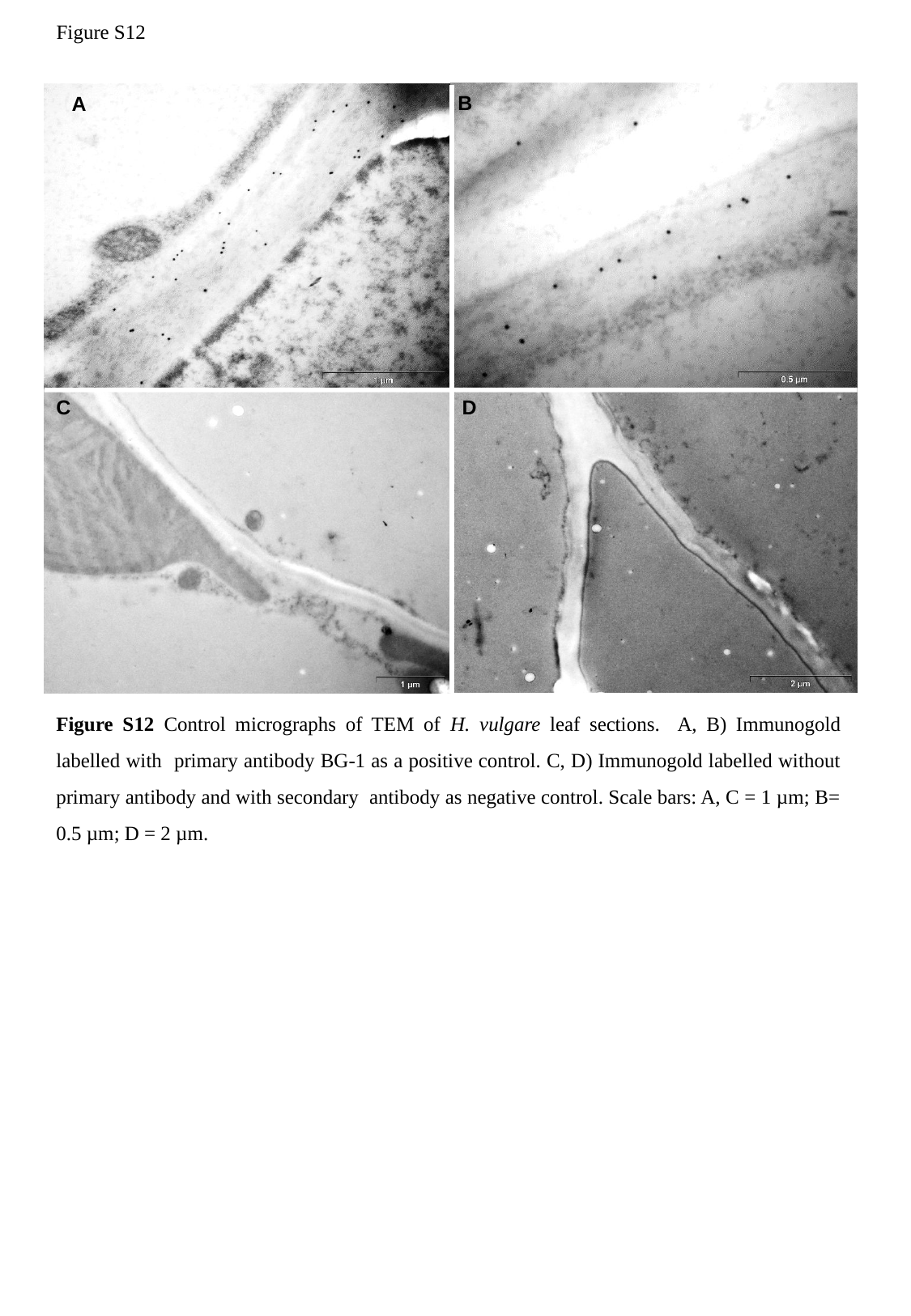

Figure S12
B
A
D
c
Figure S12 Control micrographs of TEM of H. vulgare leaf sections. A, B) Immunogold labelled with primary antibody BG-1 as a positive control. C, D) Immunogold labelled without primary antibody and with secondary antibody as negative control. Scale bars: A, C = 1 µm; B= 0.5 µm; D = 2 µm.
