# Supplementary material for: Genetics and physiology of cell wall polysaccharides in the model C4 grass, Setaria viridis spp
Source: BMC Plant Biol. 2015 Oct 2;15:236. doi: 10.1186/s12870-015-0624-0 (PMC4592572; doi:10.1186/s12870-015-0624-0)
Supplement: Additional file 2: Table S1. — Cellulose synthase (CesA) and Cellulose synthase-like (Csl) gene families in S. italica. Table S2. Primer sequences for the amplification of transcripts of control genes and Cellulose synthase-like genes (Csl) F, H and J in S. viridis. (DOCX 19 kb) [file 12870_2015_624_MOESM2_ESM.docx]

**Supporting Tables**

**Table S1 *Cellulose synthase* (*CesA*) and *Cellulose synthase-like* (*Csl*) gene families in *S. italica*.**

| Family | Genes | Gene ID | Chromosome |
| --- | --- | --- | --- |
| *CesA* | *CesA1* | Si028770m | 2 |
|  | *CesA2-1* | Si005779m | 4 |
|  | *CesA2-2* | Si028762m | 2 |
|  | *CesA2-3* | Si028764m | 2 |
|  | *CesA2-4* | Si034009m | 9 |
|  | *CesA3* | Si034016m | 9 |
|  | *CesA4* | Si034020m | 9 |
|  | *CesA5* | Si000179m | 5 |
|  | *CesA6-1* | Si000133m | 5 |
|  | *CesA6-2* | Si005742m | 4 |
|  | *CesA6-3* | Si021050m | 3 |
|  | *CesA8* | Si028761m | 2 |
|  | *CesA10* | Si007846m | 4 |
| *CslD* | *CslD1* | Si015820m | 6 |
|  | *CslD2* | Si005721m | 4 |
|  | *CslD3* | Si033974m | 9 |
|  | *CslD4* | Si021011m | 3 |
|  | *CslD6* | Si008500m | 4 |
| *CslE* | *CslE-1* | Si029057m | 2 |
|  | *SclE-2* | Si029066m | 2 |
| *CslF* | *CslF3-1* | Si031960m | 2 |
|  | *CslF3-2* | Si032230m | 2 |
|  | *CslF4* | Si028885m | 2 |
|  | *CslF6* | Si013204m | 6 |
|  | *CslF7* | Si034259m | 9 |
|  | *CslF8* | Si028860m | 2 |
|  | *CslF9* | Si028873m | 2 |
| *CslH* | *CslH-1* | Si009413m | 7 |
|  | *CslH-2* | Si016419m | 1 |
|  | *CslJ-1* | Si021430m | 3 |
| *CslJ* | *CslJ-2* | Si024965m | 3 |

**Table S2 Primer sequences for the amplification of transcripts of control genes and *Cellulose synthase-like* genes (*Csl*) *F*, *H* and *J* in *S. viridis*.**

| Gene | Forward Primer | Reverse Primer | PCR size bp | Tm°C |
| --- | --- | --- | --- | --- |
| *GAPSv* | GAGTTGCCTTTTGCTTTTCCT | GCGGGACAAAACATGAAACTA | 189 | 80 |
| *ELFSv* | AAGAACGGTGATGCTGGTATG | GTCCTTCTTCTCCACGCTCTT | 153 | 83 |
| *ActinSv* | TGTGCTCAGCGGTGGCTCAAC | AGGGAGGCAAGGATGGACCC | 156 | 83 |
| *Tub1Sv* | GTTCCGGAGGGTGAGCGAGC | GCGGTCGCGTCCTGGTACTG | 160 | 84 |
| *CslF4Sv* | AAGAAACCTGCCATCCTGTTT | TGTGCAATGGCATTATCAAAA | 184 | 80 |
| *CslF6Sv* | AAGTTCGGTTACTCGGAGGTC | TCACCAGCAGCAGGTAGAAAT | 154 | 83 |
| *CSLF7Sv* | TGACGTTGAGCCCACTACTCT | GTCACCAATCTTGTGCTGGTAA | 224 | 79 |
| *CslF8Sv* | TGATCTACCCCTTTGCACTTG | TGTGCCACTACTCAACCACAA | 216 | 83 |
| *CslF9Sv* | CAGAGAGCTGGAGGCGGATTT | TGTGTGGTTTTTATCCCCATCTGA | 186 | 78 |
| *CslH1Sv* | CAAGATCAAGGCTGTTCTGCT | TCCGAACTCAGATTCTTCTGC | 224 | 82 |
| *CslH2Sv* | GTGTTGGAGAATTCGTGTGCT | ACGCTGCGAAAGTTAAGTTCA | 170 | 82 |
| *CslJ2Sv* | GCAGCTGTTTCTCCTGAGCTA | TAGCTTTTTGCACTCCATCGT | 206 | 85 |
